# Electromagnetic Functions Modulation of Recycled By-Products by Heterodimensional Structure

**DOI:** 10.1007/s40820-025-01659-7

**Published:** 2025-02-06

**Authors:** Ze Nan, Wei Wei, Zhenhua Lin, Ruimei Yuan, Miao Zhang, Jincheng Zhang, Jianyong Ouyang, Jingjing Chang, Hejun Li, Yue Hao

**Affiliations:** 1 State Key Laboratory of Wide-Bandgap Semiconductor Devices and Integrated Technology, School of Microelectronics, Xidian University, Xi’an, 710071 People’s Republic of China; 2 State Key Laboratory of Solidification Processing, Shaanxi Province Key Laboratory of Fiber Reinforced Light Composite Materials, Carbon/Carbon Composites Research Center, Northwestern Polytechnical University, Xi’an, 710072 People’s Republic of China; 3 Advanced Interdisciplinary Research Center for Flexible Electronics, Xidian University, Xi’an, 710071 People’s Republic of China; 4 Department of Materials Science and Engineering, National University of Singapore, 7 Engineering Drive 1, Singapore, 117574 Singapore

**Keywords:** Silver nanowires, Electromagnetic shielding, Microwave absorption, Recycled aerogels

## Abstract

One of the significant technological challenges in safeguarding electronic devices pertains to the modulation of electromagnetic (EM) wave jamming and the recycling of defensive shields. The synergistic effect of heterodimensional materials can effectively enable the manipulation of EM waves by altering the nanostructure. Here we propose a novel approach for upcycling by-products of silver nanowires that can fabricate shape-tunable aerogels which enable the modulation of its interaction with microwaves by heterodimensional structure of by-products. By-product heterodimensionality was used to design EM-wave-jamming-dissipation structures and therefore two typical tunable aerogel forms were studied. The first tunable form was aerogel film, which shielded EM interference (EMI shielding effectiveness (EMI SE) > 89 dB) and the second tunable form was foam, which performed dual EM functions (SE > 30 dB& reflective loss (RL) < -35 dB, effective absorption bandwidth (EAB) > 6.7 GHz). We show that secondary recycled aerogels retain nearly all of their EM protection properties, making this type of closed-loop cycle an appealing option. Our findings pave the way for the development of adaptive EM functions with nanoscale regulation in a green and closed-loop cycle, and they shed light on the fundamental understanding of microwave interactions with heterodimensional structures.

**Supplementary Information:**

The online version contains supplementary material available at 10.1007/s40820-025-01659-7.

## Highlights

- Turning trash into treasure: The outstanding tunable aerogels were fabricated via heterodimensional by-products of silver nanowires. The first tunable form, aerogel film, shields electromagnetic interference (EMI SE > 89 dB), while the second tunable form, aerogel foam, performs dual EM functions (EMI SE > 30 dB and RL < -35 dB, EAB > 6.7 GHz).
- Recycle again: The secondary recycled aerogels retain nearly all of their EM protection qualities, making this closed-loop cycle desirable.

**Supplementary Information:**

The online version contains supplementary material available at 10.1007/s40820-025-01659-7.

## Graphical abstract

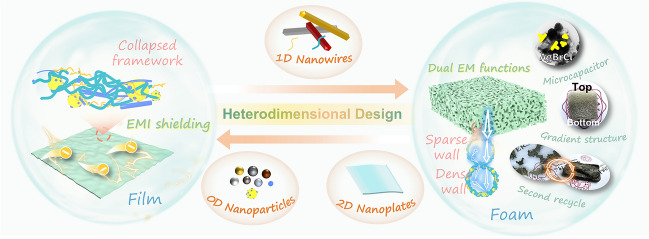

## Introduction

With the continuous scaling of complementary metal–oxide–semiconductor (CMOS) technology, three-dimensional (3D) integration has emerged as a practical solution to enhance device density by vertically stacking devices or dies, keeping pace with Moore’s law [1, 2]. However, this stacking approach significantly exacerbates electromagnetic interference (EMI), which poses a critical challenge to the reliability and stability of integrated circuits [3–5]. Beyond compromising circuit performance, the increasing intensity of EMI generated by high-density integrated systems can also extend its impact to human health and the surrounding environment, if no shielding is provided [6–9]. Presently, nanomaterial-based EM-functional shrouds are appealing due to their ultrahigh conductivity, flexibility, and varied dimensions [10]. Yet, how to deal with themselves and their by-products, however, is challenging because those are probably nanotoxic (including carcinogenicity and reproductive toxicity) and degradation-resistant [11–14]. In addition, the growing environmental impacts associated with electronic waste make sustainable development in next-generation consumer electronics essential [15, 16].

Low-dimensional nanomaterials for EM function contain different dimensions (zero-dimensional (0D), quasi-one-dimensional (Q1D), one-dimensional (1D), and two-dimensional (2D) nanofillers) [17–19]. The ingenious combination of these different dimensions known as heterodimensional structures will exhibit novel physical properties (containing nanostructure, conductive network distribution, EM response, etc.) beyond those of the parent material at the nano/micro/macroscale [20]. The modulation of heterodimensional structure can effectively realize super effective EMI shielding or better microwave absorption (MA) and, to some extent, enables the integration of EM dual functions, specifically EMI shielding and MA [17, 21, 22]. For instance, the synthesis of 1D metal nanowires (MNWs)/carbon nanotubes (CNTs) and 2D graphene/MXene boosts the reflective dissipation and enhances the flexibility of the whole system [23–26]. Likewise, the combination of 0D nanomagnet and 1D/2D nanoconductor in a single composite can synchronously achieve the “conductive-magnetic” dual loss and strong attenuation for consuming the incident wave, ultimately contributing to the excellent EM dissipation performance [21]. Nevertheless, the advancement of a heterodimensional system for EM shrouds encounters significant challenges. These obstacles encompass understanding the structure–function relationship among heterodimensional nanomaterials, striking a balance between high conductive loss and impedance match, and determining an effective approach for recycling the nanofillers involved [17, 27–29].

Silver nanowires (AgNWs), owing to their ultrahigh length-to-diameter ratio, marvelous intrinsic stretchability, and champion bulk conductivity of metal, have emerged as promising EM function materials with a high EMI SE and the superpower to produce ultralight free-standing porous aerogel [30–32]. Currently, most AgNWs are synthesized using a solution-phase method, which produces numerous by-products that include silver halide particles and other-sized nanosilvers (including 0D Ag nanoparticles (AgNPs), Q1D Ag nanorods (AgNRs), short 1D AgNW (< 10 ~ 20 μm) and other solids with indistinguishable dimension) [33–35]. Surprisingly, this by-product system containing multidimensional solids also introduces a large number of poorly conductive silver halide MA clusters for impedance matching, which also can help preserve the EM response characteristics of highly conductive nanosilver. Hence, the combination of 0D silver halide absorbers and 0/Q1/1D shields renders the by-product system unique in its response to incident EM waves and potentially offer a possibility to regulate EM response behavior.

Lightweight aerogels, with their high specific surface area and excellent mechanical, electrical, and thermal properties, hold great promise for EM protection applications [36]. However, traditional aerogels are typically composed of materials with limited dimensional diversity, which constrain the versatility of their conductive networks. The freeze-drying technique offers an effective method for the assembly of heterodimensional by-products into aerogel frameworks, enabling the design of dual EM-functional aerogels with more sophisticated conductive networks. Also, this approach facilitates the enhancement of EM dissipation properties, thus expanding the potential of aerogels for advanced EM protection applications. To date, no heterodimensional AgNWs by-products-based aerogel have been reported in the literature.

**Fig. 1:**
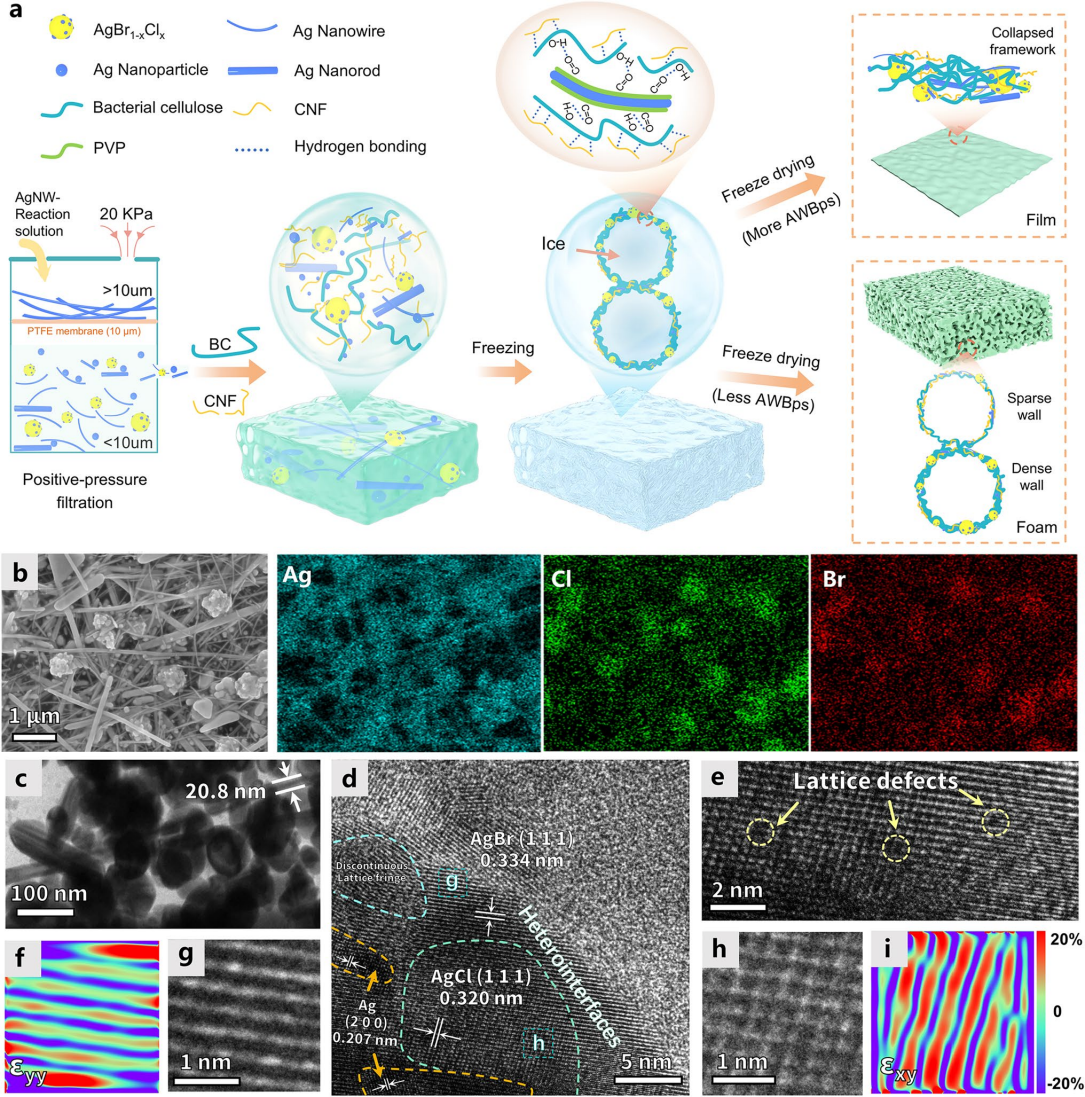
Schematic and morphology of MC-assisted AWBps-based aerogels. **a** Schematic of the freeze-drying preparation process for MC-assisted AWBps-based aerogels. **b** SEM and EDS elemental mapping of representative AWBPs. **c** TEM image of AWBps. **d** HRTEM of AgBr_x_Cl_1-x_ in **c**. **e, g, h** Magnified HRTEM images of the sample shown in **d** (regions **g, h**), revealing the lattice defects, and discontinuous lattice fringe. **f, i** The corresponding strain fields ϵ_yy_ were determined by the GPA method using the TEM image **g, h**, respectively

Herein we present an effective method for upcycling AgNWs by-products (AWBps) that can fabricate the resultant aerogel which enables the modulation of its interaction with microwaves by heterodimensional structure of AWBps. We systematically revealed the morphology of aerogel (containing the spatial relation of these heterodimensional nanofillers and macroscopic aerogel shapes) in various proportions of nanoparticles and nanowires (Fig. 1a), as well as the resulting disparate EM response. Additionally, the heterodimensional characteristics of by-products are strategically used to devise unique structures (including polymorphic and multiscale pores, large nanoparticle agglomeration-induced localization of conductive network (in the horizontal direction) and conductive gradient (in the vertical direction)) that contributed significantly to EM wave dissipation. Remarkably, benefiting from the above synergistic effects, the resultant aerogel film and foam show tunable EM protective function on Tunable form I and Tunable form II, demonstrating outstanding EMI SE or dual EM protective function, respectively. The demonstrated control of reflection and absorption of EM waves by heterodimensional nanofillers provides a realistic approach toward adaptive EM protection. Furthermore, the cellulosic AWBps aerogel can be easily recycled by mechanically disintegrating the product at its end-of-life to reform a uniform AWBps-cellulose slurry for reuse. In contrast, the secondary recycled aerogels exhibit comparable EM protection capabilities, thereby showcasing an appealing closed-loop cycle characteristic. This flexible, effective EM dual protective, and recyclable cellulosic AWBps aerogel with a substantially reduced AgNWs waste footprint demonstrates herein is a strong candidate for replacing widely first-time used EM shrouds toward changing electronics application (for example, IC package, transient electronics, artificial skin).

## Experimental Section

### Materials

AgNO_3_ and polyvinylpyrrolidone (PVP, MW = 55,000, 360,000) and Ethylene glycol (EG) were supplied from Adamas-beta (China). NaCl and NaBr were purchased from General Reagent (China). Bacterial cellulose (BC) and cellulose nanofibrils (CNF) suspension were obtained from Guilin Qihong Technology (China). All reagents were used as-received without further purification.

### Preparation of AWBps Aerogel

#### Synthesis of AgNW

AgNW was synthesized according to our previous work. 0.220 M NaBr, 0.210 M NaCl, and 0.505 M PVP (MW = 360,000) in EG were individually prepared for utilization. Fresh AgNO_3_ was dissolved in EG by sonicating in an ice bath (4–8 °C) for 5 min. Subsequently, 2.5 mL of NaBr, 5 mL of NaCl, 25 mL of PVP and 25 mL of AgNO_3_ (0.265 M) were successively added to a 100 mL flask placed in an oil bath at room temperature. Vigorous stirring was applied for 30 min and then the temperature in the flask was elevated to 170 °C in 15 min, where the nitrogen gas with a flux of 150 mL min^−1^ was used to cover the liquid surface, creating an oxygen-free atmosphere. Thereafter, the flask was corked and the reaction was left for 1 h without disturbing. The flask was taken off from the oil bath immediately and transferred to the water for cooling once the reaction was terminated.

#### Purification of AgNWs

The hydrophilic polytetrafluoroethylene (PTFE) membrane (diameter: 100 mm, pore size: 10 μm) was treated with 70 °C de-ionized (DI) water for 30 min and then installed in a mass-filtration system. A polypropylene (PP) membrane (diameter: 100 mm, pore size: 10 μm) was used as the supporting layer to prevent damage to the PTFE membrane. Next, 15 mL of the AgNW reaction solution was mixed with 35 mL of DI water, and the mixture was transferred to a positive-pressure filtration system. Filtration was performed under a pressure of 5–20 kPa, provided by an air compressor. The AgNWs remaining on the membrane were then redispersed into 30 mL of 1wt% PVP solution (MW = 55,000) under vortex mixing for 3 s. Finally, the AgNW by-products were collected by centrifuging the filtrate at 5000 rpm for 12 min. The resulting AgNW by-products (AWBPs) were then redispersed into DI water at various concentrations.

#### Fabrication of AWBps-Based Aerogels

First, a mixture of BC (0.8 wt%) and CNF (1 wt%) was stirred homogeneously according to equal volume ratio. Afterward, a series of AWBPs dispersions with different contents (1 ~ 5 wt%) was added into the mixture of cellulose suspensions (MC), and stirring continued for 15 min to obtain a homogeneous mixture of MC/AWBps-*x* (*/*AgNW-*y*) suspensions, where *x* represents the filler content of AWBps (and *y* represents that of AgNWs). After the suspension was frozen entirely, the frozen gel was kept in the refrigerator for 12 h. Then, the sample was freeze-dried for 12 h to attain the resultant aerogel film/foam in a freeze dryer (10 Pa, − 60 °C). Note that the suspension with AgNWs added corresponds to the aerogel foam. For comparison, AgNWs/MC, large Ag nanoparticles (L-AgNPs)/MC and small Ag nanoparticles (S-AgNPs)/MC aerogels were prepared in the same steps as above.

### Reutilization of Secondary Recycled AWBps-Based Aerogel

To test the recycling capability, the shredded MC/AWBps aerogels were immersed in DI water and then stirred until the whole aerogel was dissolved at room temperature. Next, AWBps were attained by collecting the sediment from the filtrate after centrifugation at 3000 rpm for 8 min. The AWBps-based colloidal suspension with controlled concentration (2.5 wt%) was obtained after redispersion in DI water. Moreover, recycled MC/AWBps/AgNWs aerogel foams were fabricated in the same steps as above.

## Results and Discussion

### Material Synthesis and Characterization

AWBps-based aerogels are prepared with the mixture of cellulose (MC, containing bacterial cellulose and cellulose nanofibrils)-assisted preparation process (Fig. 1a). Firstly, the addition of NaBr and NaCl in the polyol synthesis of AgNWs in ethylene glycol (EG) results in the rapid formation of AgBr_x_Cl_1-x_ nanocuboids, which induces the metallic Ag to nucleate unevenly on their surfaces [37]. Subsequently, AgNWs grow from these nucleation sites. After the reaction, AWBps dispersion (containing the residual AgBr_x_Cl_1-x_ nanocuboids and the nanosilver not formed as long AgNWs) is collected after the purification of AgNWs by positive-pressure filtration. During filtration, long silver nanowires are deposited on the filter membrane (Figs. S1, S2), and particles smaller than the pore diameter of the filter membrane are filtered, which is generally discarded as waste. As shown in Fig. S3, a UV–vis spectrum of the filtrate shows stronger absorption in the bands of 400–500 nm corresponding to more nanoparticles in it [38]. The SEM images, corresponding element maps and transmission electron microscopy (TEM) point out that the by-products are composed of a handful of silver halide particles (containing AgBr_x_Cl_1-x_ clusters, etc.) as well as plenty of nanosilvers (including AgNPs, AgNRs, short AgNWs) (Fig. 1b, c). The enlarged TEM image of AgBr_x_Cl_1-x_ depicts that the randomly synthesized silver crystals in the surface of silver halide will co-act for “Ag-AgBr_x_Cl_1-x_-Ag” nanocapacitors, which exhibit high charge storage capacities, thereby intensifying the polarization loss of incident EM waves (Fig. S4) [39].

Meanwhile, the atomic compositions detected by EDS indicated that the Ag element is dominant, which results in a strong conduction loss when EM waves pass through the materials (Figs. S5 and S6). According to the high-resolution transmission electron microscopy (HRTEM) image (Fig. 1d), AgBr_x_Cl_1-x_ was composed of face-centered cubic Ag, AgBr and AgCl (Fig. 1d), whose mutual boundaries were observed. Ulteriorly, numerous structural defects, including discontinuous lattice fringes and point defects, were clearly observed from the part of TEM and the enlarged HRTEM images (Fig. 1d, e, g, h). Quantificationally, the lattice strain (Fig. 1f, i) was calculated using the geometric phase analysis (GPA) method, and the corresponding strain mapping revealed a high strain concentration [40]. Specifically, GPA analyses showed that both axial strain (ϵ_yy_, which can be compressive or tensile strains) and shear strain (ϵ_xy_) are distributed along lattices of AgBr_x_Cl_1-x_ and reach a maximum value of ϵ_yy_ =  ~ 20% (Fig. 1f) and ϵ_xy_ =  ~ 20% (Fig. 1i) [41]. The “strain induced-defect-rich” characteristics endow AgBr_x_Cl_1-x_ clusters with strong EM wave attenuation [42, 43].

### Heterodimensional Structure Modulated EM Function of Aerogel

**Fig. 2:**
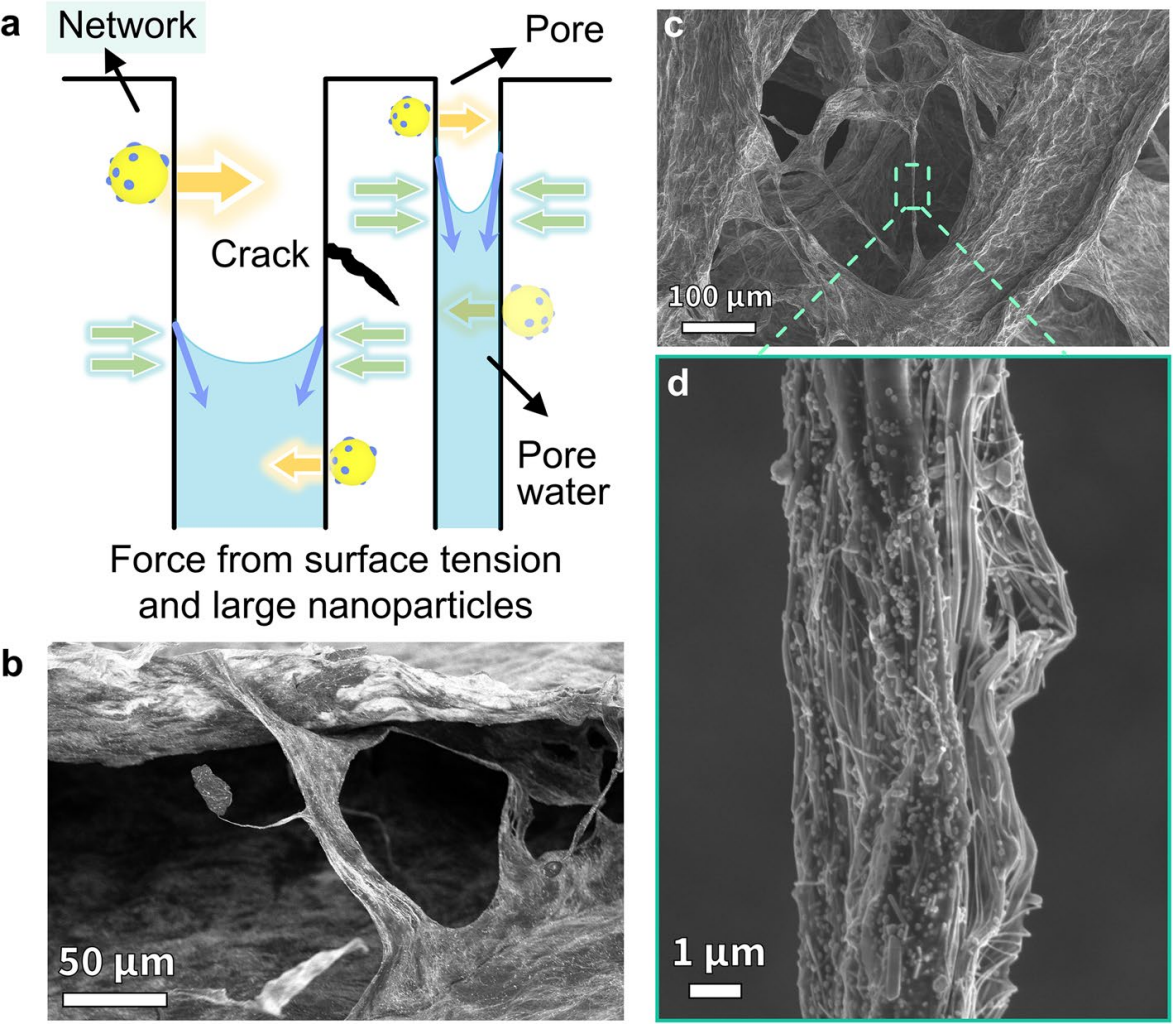
Diagram of external force on the forming of aerogel foam/film and the related images of "network" in the diagram. **a** Schematic of capillary forces and large nanoparticles inducing cracks or shrinkage in the pores of the gels. **b** Enlarged SEM image of the nacre-like/bidirectional structure shown in Fig. S9, revealing the connection structure of layers. **c** SEM images of AWBps/AgNWs/MC aerogel foam. **d** Enlarged SEM image of **c**, showing the connecting filament

After the AWBps were collected, we employed the simple freeze-drying technology to form two different aerogels with the help of capillary force differences in two heterodimensional structures. These aerogels are defined as *Tunable form I: film* and *Tunable form II: foam* (Fig. 1a). Notably, the heterodimensional structure modulated aerogel shape (film/foam) can further effectively change the EM response (Text S1), thereby completing the EM function transformation. Simply put, the external force of 0D large particles and the surface tension caused by obstructing the heat dissipation of the system will cause aerogel to densify and tend to be thin films in form (Figs. 1a and 2a). This makes the conductive network more centralized, and conducive to efficient EMI shielding. Whereas, the addition of a small amount of heat-dissipating 1D AgNWs weakens the capillary force and forms the connection filaments (Figs. 2b-d and S7-S9), causing the aerogel morphology to favor foam (Figs. S10 and S11). The foam itself will improve the impedance matching compared with the film, and can provide multiple reflection planes as well, which makes the overall more conducive to MA.

#### EMI Shielding Performance of AWBps Aerogel Film (Tunable Form I)

**Fig. 3:**
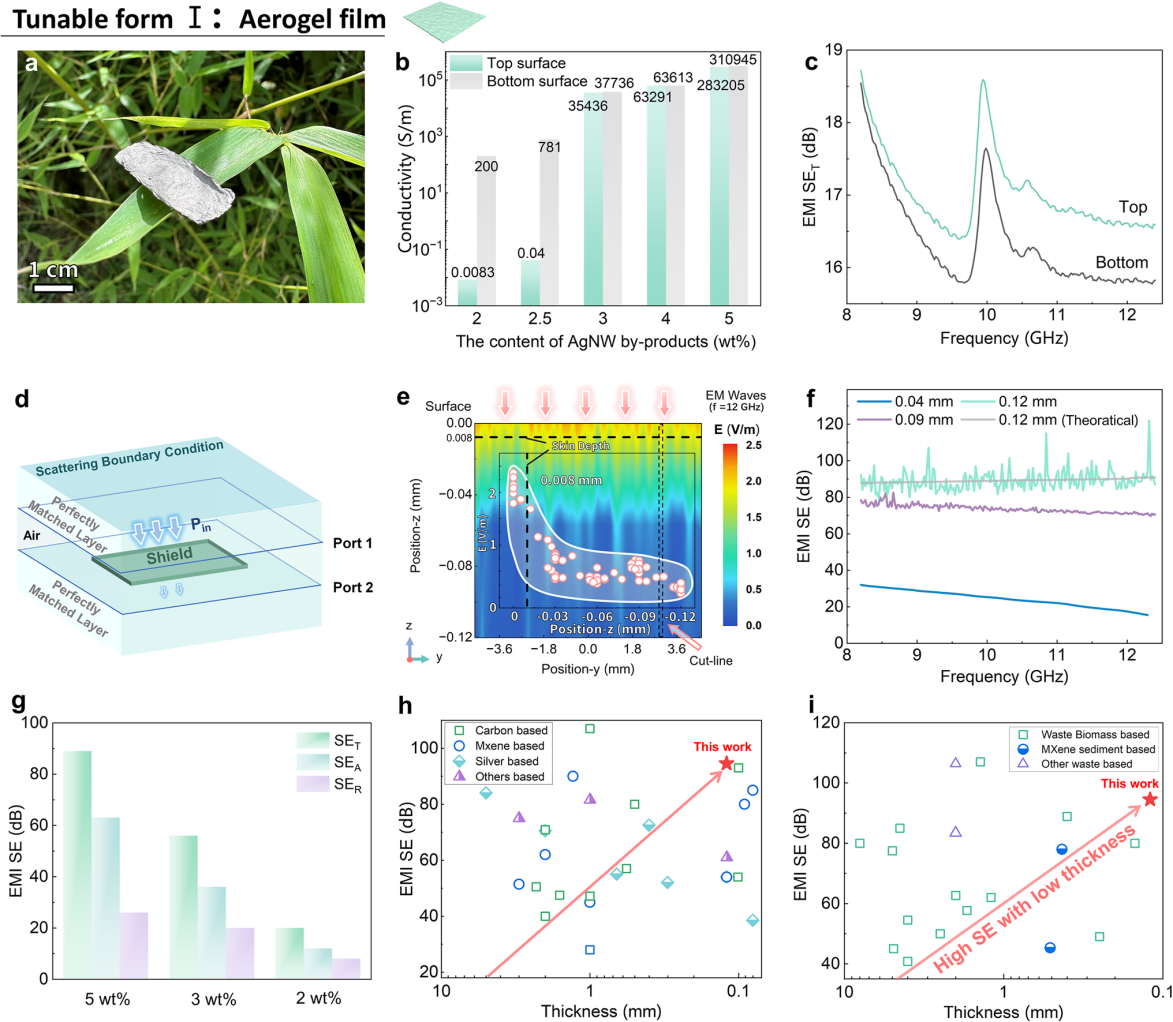
EMI shielding behaviors of AWBps aerogel films. **a** Digital photo of aerogel film. **b** Electrical conductivity of the aerogel films of top and bottom surfaces with various mass ratios of AWBps. **c** EMI SE of 2 wt% AWBps aerogel film at a thickness of 0.12 mm when EM wave incidence from the top or bottom surface. **d** Schematic diagram of FEA simulation. **e** FEA simulated the electric field intensity of aerogel film and the distribution of its cut-line, particularly showing its skin depth at 0.008 mm in the z-position. **f** EMI SE of 5 wt% AWBps aerogel film at different thicknesses. **g** Total average EMI SE (SE_T_) and its absorption (SE_A_) and reflection (SE_R_) mechanism with various mass ratios of AWBps. EMI SE versus thickness of **h** different nanomaterials and **i** waste materials (the best results for the frequency bands given in the literature). A detailed description of each data point is presented in Tables S3 and S4

Benefiting from this multiscale cellulose-based matrix (Text S2, Figs. S12 and S13), an ultrathin and dense AWBps aerogel film is fabricated with a high mass ratio of AWBps nanofillers (Figs. 3a and S14). Effective composite of MC and AWBps is also confirmed by the presence of the cellulose (110) (200) peaks in the X-ray diffraction (XRD) patterns of MC/AWBps aerogel (Fig. S15). Thanks to the tough multiscale cellulose structural matrix, AWBps aerogels can be bent with any curvature and no cracks or fractures are observed (Fig. S16, Tables S1, S2), showing superior flexibility.

High EMI SE values typically require the use of materials with a high electrical conductivity [3]. A typical current–voltage (*I − V*) curve of the AWBps aerogel at room temperature is shown in Fig. S17, indicating AWBps aerogel as a pure resistance. In addition, Fig. 3b presents the electrical conductivity of the aerogel films of top and bottom surfaces with various mass ratios of AWBps. With the addition of only 2 wt% AWBps, the conductivity of aerogel film rises to 0.0083, 200 S m^−1^ for top and bottom surfaces, respectively. The reason for the sharp difference in upper and lower conductivity can be mainly summarized as the uneven distribution of upper and lower nanosilvers due to more low-migration-velocity-induced deposition of “AgBr_x_Cl_1-x_ large particles-nanosilvers” aggregates during the migration of pore fluid toward a crystalline layer on the surface of the gel particularly under low fillers [44, 45]. As filler content is increased, the conductivity increases and reaches 283,205, 310,945 S m^–1^ for the 5 wt% AWBps composite of top and bottom surfaces, respectively. This proves that many nanosilvers can be completely dispersed in the unit spatial medium at microscale thickness, making the upper and lower electrical conductivity uniform. Such excellent electrical conductivity results from significant increase of long-range conductive paths of AgNWs and local current shortcuts of AgNPs/AgNRs (Fig. S18), making this nanofiller-based film bulk silver-like in nature.

To verify the conductive gradient structure effectiveness for shielding, EMI SE of 2 wt% AWBps film was measured from the top and bottom direction, showing ~ 16.5 and ~ 15.8 dB, respectively (Figs. 3c and S19a). Top incidence can effectively increase EMI SE, blocking 97.8% of incident radiation, by “less reflection on the surface, more absorption in the shields” strategy (Fig. S19b) [46]. To further explore the superiority of AWBps film, finite element analysis (FEA) was conducted to calculate the EMI SE and electric field based on the standard parameters of each component (Fig. 3d-f). The vertical cross-section electrical field distribution of AWBps film (5 wt%, 0.12 mm) shows that the charge is mainly located at the skin depth (0.008 mm) down from the top surface due to skin effect (Fig. 3e, insert scatter plot). As the EM waves travel through the interior of the film, the electrical field weakens, indicating that the EM waves lose energy. Since thickness is so important to EMI SE, increasing material thickness is a simple way to boost EMI SE. To investigate this effect, we measured the EMI SE of three AWBps films with different thicknesses in Fig. 3f. The ultrahigh EMI SE value shows 89.1 ± 5.4 dB for a 0.12-mm-thick film (even reaches 122 dB at 12.3 GHz), enough to block 99.9999999% of incident radiation. Experimental results of an AWBps film in the X-band are comparable to the theoretically calculated values using FEA that predicts high EMI SE values of various mass content or thickness as well.

Furthermore, AgNWs and AgNRs in AWBps, with a high length-to-diameter ratio, naturally facilitates the formation of highly efficient shielding networks under low fillers. To better ascertain the important influences of AWBps content, we investigated the AWBps composites with various filler contents for EMI shielding. Here, the thickness of composite films was fixed around 0.12 mm. With increasing AWBps content, EMI SE increased sharply (Fig. 3g). We also found that the shielding due to absorption was the dominant mechanism, rather than reflection in AWBps composite films. Figure 3h shows the comparison of EMI SE versus thickness for different nanofiller-based EMI shielding materials. The AWBps composite film prepared in this work achieves high EMI SE (89.1 ± 5.4 dB) with a small thickness of 0.12 mm, which is superior to similar materials reported (Table S3). Simultaneously, we compared the recently reported EMI shielding performance of upcycling shields following the “waste-to-wealth” strategy, as shown in Fig. 3i. A comprehensive literature review of previously studied upcycling materials for EMI SE (Table S4) clearly indicates that AWBps composites are the best EMI shielding materials from “waste” known to date. This result also proves the high-probability feasibility of AWBps upcycle and the marvelous effectiveness of recycling methods.

#### Dual EM Function of AWBps Aerogel Foam (Tunable Form II)

**Fig. 4:**
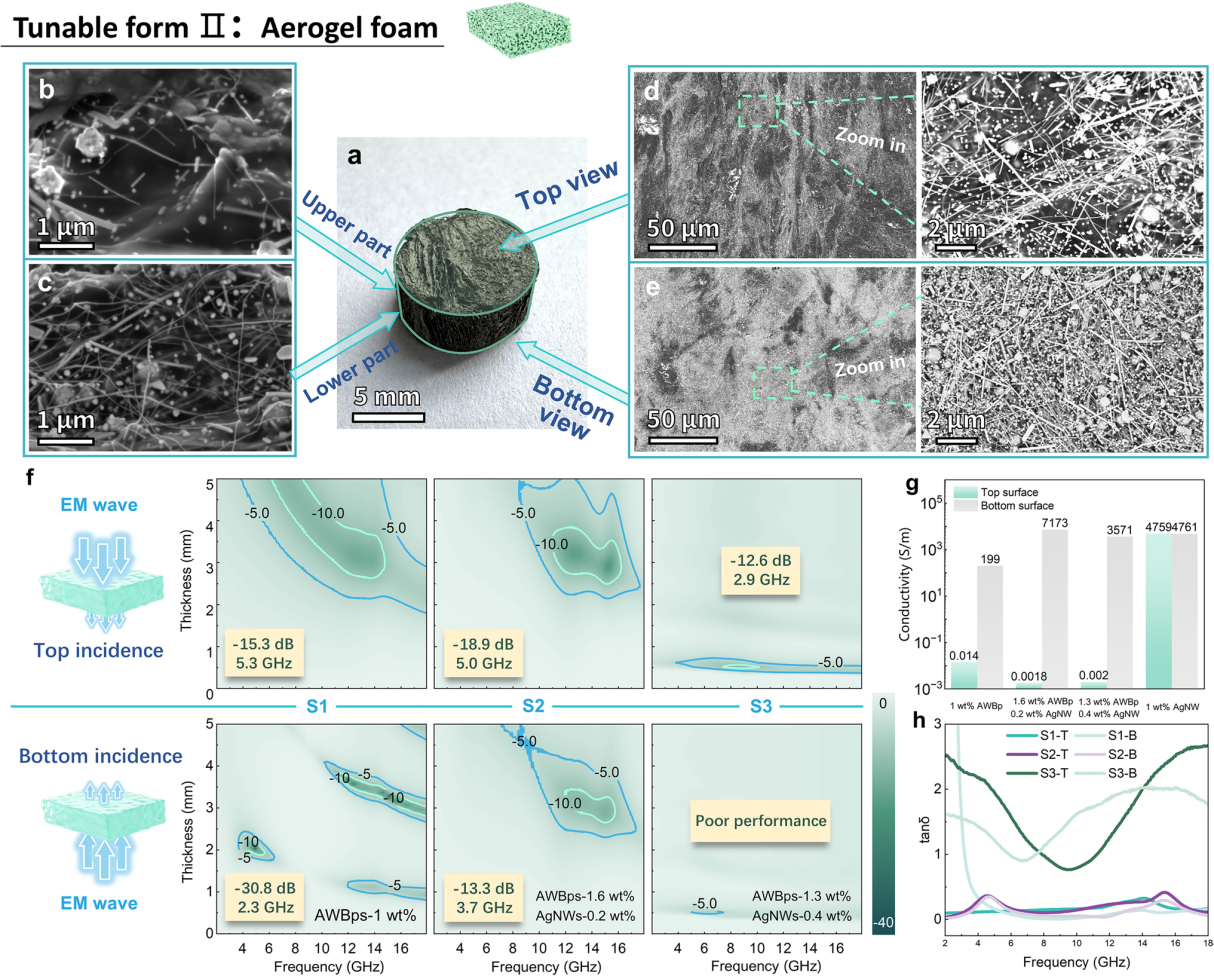
Microwave absorption behaviors of AWBps/AgNWs aerogel foams. **a** Digital photo of aerogel foam. SEM images of **b** upper part of foam, **c** lower part of foam, **d** top view, and **e** bottom view. **f** 2D RL plots of 1 wt% AWBps, 1.6 wt% AWBps-0.2 wt% AgNWs and 1.3 wt% AWBps-0.4 wt% AgNWs aerogel foam, containing top incidence and bottom incidence modes. **g** Electrical conductivity and** h** dielectric tangent loss of the aerogel foams of top and bottom surfaces with various mass ratios of AWBps-AgNWs

According to percolation theory, AWBps-only (impurity-dominated) aerogel makes it hard to form a full foam structure. Experimentally, at low AWBps concentrations, aerogel can only be formed tentatively and incompletely even with a multiscale cellulose matrix due to capillary stress [47]. Compared with cellulose matrix, AgNWs matrix owns higher thermal conductivity, allowing it to tolerate and utilize more AgBr_x_Cl_1-x_ nanocapacitors (beneficial for EM dissipation), thereby enhancing overall conductivity loss. Hence, adding a small amount of AgNWs can effectively transform into AgNWs-cellulose dominated heterodimensional system, thereby reducing the difficulty of thickness-direction formation and promoting the formation of porous foam. The AWBps/AgNWs aerogel foam is fabricated by changing the heterodimensional nanostructure (Fig. 4a). Similarly, a foam with fewer AWBps can more easily form a conductive gradient structure because of the increased thickness for nanosilvers distribution (Fig. S20). Some SEM images for the upper part, lower part, top view, and bottom view of foam show that nanosilvers are distributed from sparse to dense from top to bottom (Fig. 4b-e).

Appropriately, the top surface has fewer nanosilvers, which greatly promotes the impedance matching and allows more EM waves to enter the absorber, while the bottom side is rich in nanosilvers with higher conductivity, which can help complete the secondary reflection of EM waves inside the material, greatly magnifying the multiple RL [46, 48]. Naturally, this unique MW absorption-promoting structure is spontaneously formed by large AgBr_x_Cl_1-x_ nanoparticles under the gravitational field, without the need for artificial additional fields such as magnetic fields [18].

To verify this gradient effect, RL is measured for three AWBps foams with different “AWBps-AgNWs” mass ratios, named S1, S2, and S3, in Fig. 4f. For AWBps-only sample (S1) with distinct gradient structure, effective MA capacity (− 15.3 dB, 5.3 GHz) is displayed for top incidence mode. But for the bottom incidence of S1, the high concentration nanosilver network results in a significant impedance mismatch (Fig. S22) and a significantly narrow EAB of 2.3 GHz. However, due to the agglomeration of large particles, there are obvious wave-transparent pores on the back side (Figs. 4e and S21), which can still allow EM waves to enter the absorber, thus achieving effective loss. For the S2, due to improved foam plumpness, AWBps/AgNWs aerogel foam exhibits effective MA performance at more AgNWs, where the *RL*_min_ is − 18.9 dB and EAB is as wide as 5.0 GHz at the matching thickness of 3.0 mm. Similar to the previous reason, the MA of bottom penetration is slightly less effective. When the AgNWs-dominated heterodimensional system comes into being, the as-prepared S3 shows poor MA performance at high AgNWs. The *RL*_min_ of − 12.6 dB and EAB of 2.9 GHz at the matching thickness of 0.6 mm are achieved because of heavy overall impedance mismatch [17, 27]. Further, normalized impedance (|Z_in_/Z_0_|) was calculated to explain the degree of impedance matching about the absorber. Generally, |Z_in_/Z_0_| in the regions of 0.8 and 1.2 should be considered as ideal impedance matching performances. For a clearer expression, the impedance values Z for all samples were given in Fig. S23. Due to the high conductivity, the impedance matching was poor. A small amount of AgNWs was added to make the foam conductive overall more uniform, which reduced the conductivity of the sample and improved the |Z_in_/Z_0_|. However, the |Z_in_/Z_0_| of S2 was both higher than 1.2, and a good impedance matching was not achieved. Electrical conductivity of the aerogel foams of S1, S2, and S3 can also verify the above reason for the difference in absorbing performance (Fig. 4g). Furthermore, the microwave loss mechanism presented in Text S3, about S1, S2, and S3 can be analyzed by the dielectric loss tangent (tan δ = ε’’/ε’, Fig. 4h) and Debye relaxation curves (also known as Cole–Cole semicircles, Fig. S22).

**Fig. 5:**
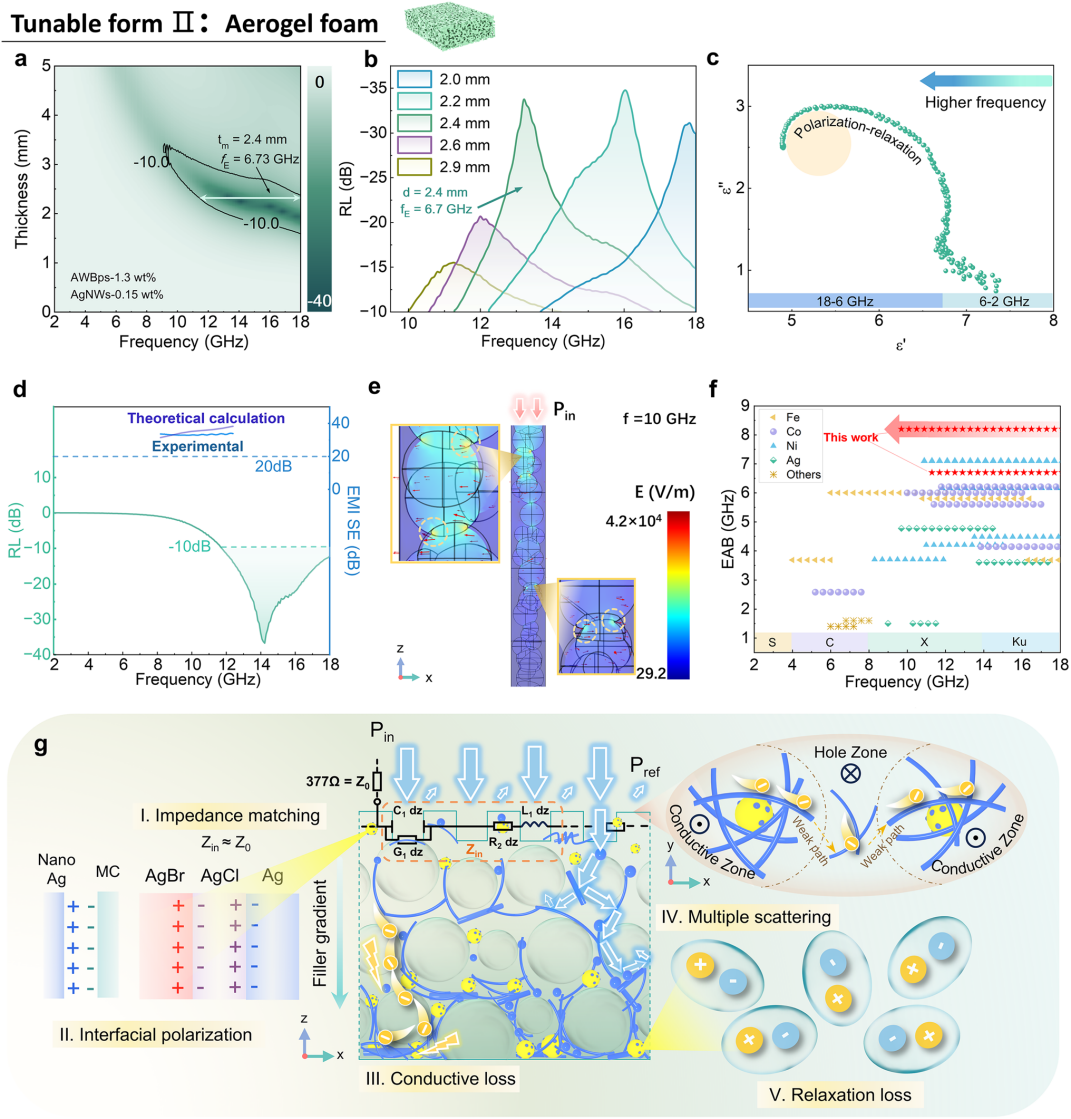
Dual EM function of AWBps/AgNWs aerogel foams. **a** 2D RL plots of post-optimized AWBps/AgNWs aerogel foam. **b** RL diagrams of the post-optimized foam with various thicknesses. **c** Cole–Cole plot of post-optimized foam. **d** EMI SE and RL plot of AWBps/AgNWs post-optimized aerogel foam. **e** Simulated electric field intensity of aerogel foam with randomly distributed pores when EM waves flood into it, revealing the presence of peak electric field. **f** Effective absorption bandwidth (EAB) at the scope of 2–18 GHz. The S, C, X, and Ku in panel **f** refer to different microwave bands, 2–4, 4–8, 8–12, and 12–18 GHz, respectively. *Source* data are presented in Table S5. **g** Schematic of the proposed EM dissipation (containing EMI shielding and MA) mechanisms for the AWBps/AgNWs aerogel foam

According to the above exploration of this heterodimensional system, it is clear that there is a tradeoff between AWBPs and AgNWs: too much AWBps reduces the overall heat dissipation performance and causes the aerogel to tend toward a thin-film form, while too much AgNWs increase the overall conductivity, further aggravating the impedance matching, and reduce the absorption performance (Text S4). Ultimately, an appropriate composed aerogel foam was prepared and its MA properties are shown in Fig. 5a, which reveals an *RL*_min_ value of − 34 dB, and EAB is as wide as 6.7 GHz, at 11.3–18 GHz (Fig. 5b). Moreover, the aerogel foam exhibited a recoverable compression strain (50%), and fatigue resistance (Fig. S24a, b and Table S2).

Based on the Cole–Cole plot derived from the dielectric constant (Figs. 5c and S24c), the AWBps/AgNWs foam displays an undulant curve containing many semicircles representing the multiple polarization relaxations, such as dipolar polarization and interfacial polarization when EM waves pass through the foam (Fig. 5c) [21, 27]. Also, the composite exhibited the best impedance matching (Fig. S25). Interestingly, the conductive gradient structure and the dispersion of AgBr_x_Cl_1-x_ shown in Figs. S20 and S26, responsible for the high absorbency also has high conductivity and corresponding conductive loss, which theoretically indicates that the foam has EMI shielding capability. Further, this excellent-MA foam has been tested for an EMI SE of up to 34 dB in the X-band (achieving 99.96% radiation attenuation), exceeding commercial standards, which means that it can realize dual EM functions (Fig. 5d) [3]. This dual EM function capacity is designed with an overall balance between conductivity (Top: ~ 0.01 S m_−1_; Bottom: ~ 500 S m^−1^) and impedance matching (Fig. S25). Obviously, if we only consider the implementation of a single EM function, it would achieve better related performance, as shown in EMI shielding film above and the recycled foam only for MA below. Additionally, peak electric fields for more dissipation of EM waves was found in a simulation of aerogel foam with randomly distributed pores subjected to an influx of EM waves (Fig. 5e).

The EABs of AWBps/AgNWs aerogel foam along with some metal-element-containing absorbers, such as ferrite, metal–organic frameworks (MOFs) and nanomagnet, are displayed in Fig. 5f and Table S5. In comparison, AWBps/AgNWs foam presents an excellent wideband EM absorption ability with EAB covering all of the Ku band and even part of X-band. Compared to the other listed, AWBps/AgNWs break the EAB limit of metal-based absorbers (especially nanosilvers) and promote the development of efficient broadband absorbers. Other dual EM-functional composites are listed in Table S6.

The aerogel foam exhibits excellent dual EM function based on the above microwave dissipation mechanism. It is concluded that the superior EM wave loss properties of the dual-EM-function AWBps-based aerogel may be principally summarized as follows (Fig. 5g): (I) Impedance matching: the existence of voids (including air voids in Figs. S27 and S28, and wave-transparent MC voids in Figs. 5d, e and S21a, b) in the surface of aerogel alters the dielectric permittivity and reduce the conductivity [21, 36], which is beneficial for impedance matching to allow more EM waves to enter the interior of absorbers; (II) Conductive loss: these losses occur as significantly induced currents are generated by the interaction between the penetrating EM waves and the high-density charge carriers (electrons and holes) within the adjacent nanosilver conductive networks, leading to the conversion of EM energy into thermal energy within the conductive pathways [9, 49, 50]; (III) Multiple scattering: The self-assembly of nanosilvers in the multiscale and hierarchical aerogel foam leads to the induction of multiple scattering of microwaves. This phenomenon results in an elongation of the travel pathway of microwaves within confined fabricated 3D complex structures (Figs. S27-S30) [51]; (IV): Polarization loss, containing interfacial loss induced by charge accumulation around the heterointerfaces owing to the different electronegativities of AgBr_x_Cl_1-x_ clusters (To prove the contribution of AgBr_x_Cl_1-x_ clusters, a nanosilvers-only aerogel (excluding AgBr_x_Cl_1-x_) was prepared and its MA properties were tested, as shown in Figs. S31-S34 and Table S7, or cellulose-nanosilvers, and strain defect-induced relaxation loss owing to the existence of numerous distorted lattices and amorphous states of metals [42, 52, 53].

### Reutilization of Secondary Recycled AWBps-Based Aerogels

**Fig. 6:**
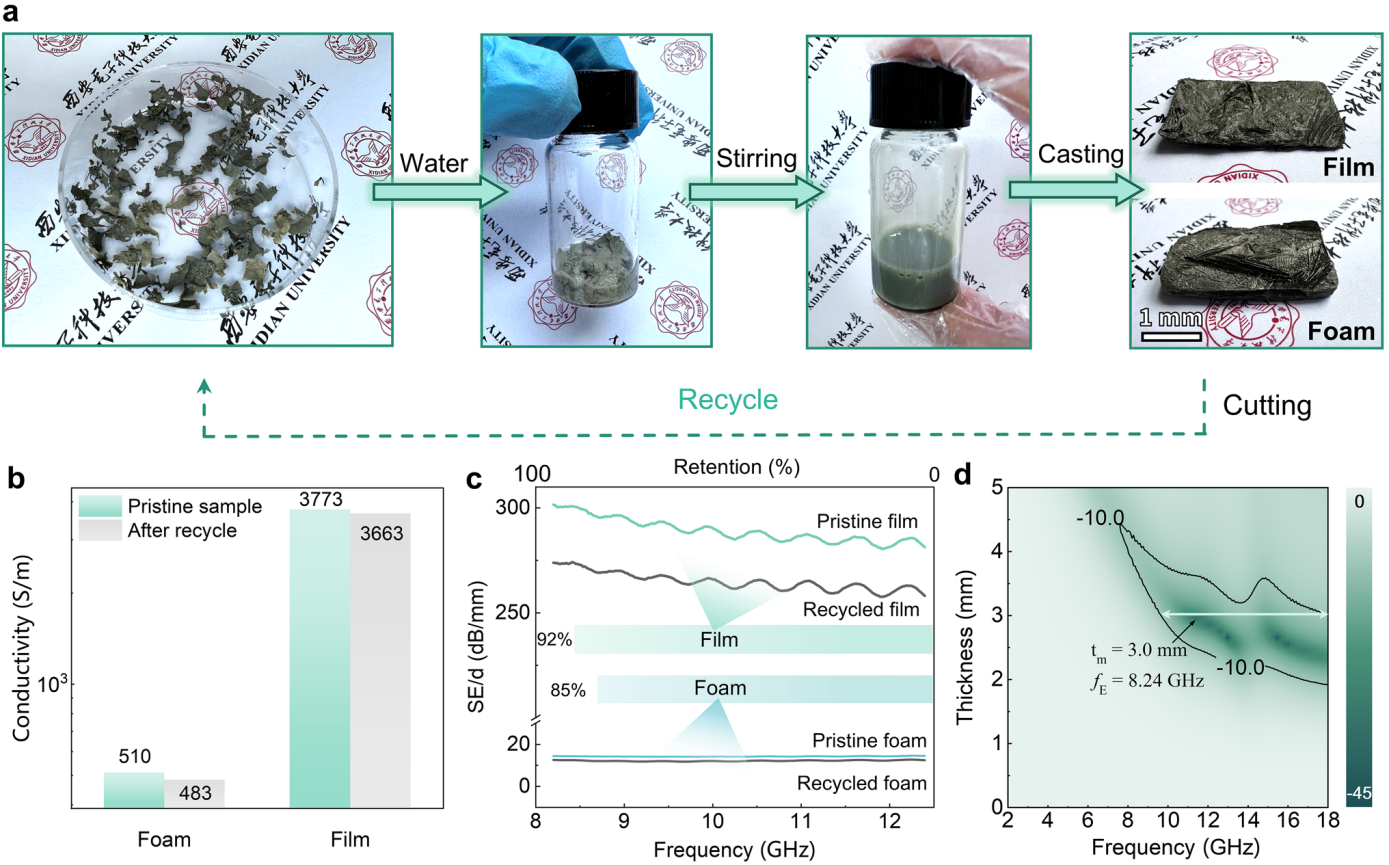
EM functions of recycled AWBps aerogels. **a** Recyclability of AWBps aerogel. **b** Electrical conductivity, **c** SE/d, and **d** *RL* of AWBps aerogel film and/or foam before and after recasting

The cellulose-assisted AWBps-based aerogels also demonstrate good recyclability. The end-of-life AWBps aerogels can be broken back down into the uniform cellulose-AWBps slurry by mechanical stirring in the ambient temperature, allowing it to be reapplied as a recycled material (Fig. 6a). This is because the cellulose/AWBps gel is crosslinked by weak physical interactions between polymer chains among celluloses and PVP through reversible interactions, e.g., van der Waals forces, hydrogen bonds and electrostatic interactions [54, 55]. However, individual nanowires have been shown to undergo some brittle failure (including bending or fracture in Figs. S35 and S36) under stirring, which can natively be explained by dislocation starvation (Text S5) [56]. Despite the partial damage, the nano-conductive network is generally intact (Fig. S37), and hence the overall conductivity and EMI SE are still basically maintained: the recycled film and foam have 92% and 85% of the original shielding capacity, respectively, as shown in Fig. 6b, c. Likewise, the effective MA capacity (-44.9 dB, 8.24 GHz) is measured for recycled foam (Fig. 6d), even showing improved MA performance after recycling due to optimized impedance matching and enhanced multiple scattering.

## Conclusions

In summary, we have demonstrated the effectiveness of AWBps upcycling approach by fabricating various EM-function aerogels which enable the modulation of their interaction with microwaves by heterodimensional structure. We systematically reveal the morphology of aerogel in various proportions of heterodimensional nanomaterials, as well as the resulting tunable EM protective function: outstanding EMI SE and dual EM protective function. The secondary recycled aerogels have almost the same capability for EM protection, providing an attractive closed-loop cycle feature. Our results offer insight into the fundamental understanding of the interactions of microwaves with heterodimensional structures and build a platform for developing adaptive EM function with dynamic regulation in a green and closed-loop cycle.

## Supplementary Information

Below is the link to the electronic supplementary material.

Supplementary file1 (PDF 4372 kb)
