# Supplementary material for: Electromagnetic Functions Modulation of Recycled By-Products by Heterodimensional Structure
Source: Nanomicro Lett. 2025 Feb 6;17:137. doi: 10.1007/s40820-025-01659-7 (PMC11803013; doi:10.1007/s40820-025-01659-7)
Supplement: Supplementary file 1 — Supplementary file1 (PDF 4372 kb) [file 40820_2025_1659_MOESM1_ESM.pdf]

Supporting Information for

Electromagnetic Functions Modulation of Recycled By-Products by Heterodimensional Structure

Ze Nan[1], Wei Wei[1, *], Zhenhua Lin[1], Ruimei Yuan[2, 3], Miao Zhang[3], Jincheng Zhang[1, 3], Jianyong Ouyang[4, *], Jingjing Chang[1, 3, *], Hejun Li[2], Yue Hao[1, 3]

[1] State Key Laboratory of Wide-Bandgap Semiconductor Devices and Integrated Technology, School of Microelectronics, Xidian University, Xi'an 710071, P. R. China

[2] State Key Laboratory of Solidification Processing, Shaanxi Province Key Laboratory of Fiber Reinforced Light Composite Materials, Carbon/Carbon Composites Research Center, Northwestern Polytechnical University, Xi'an, 710072, P. R. China

[3] Advanced Interdisciplinary Research Center for Flexible Electronics, Xidian University, Xi'an 710071, P. R. China

[4] Department of Materials Science and Engineering, National University of Singapore, 7 Engineering Drive 1, Singapore 117574, Singapore

*Corresponding authors. E-mail: weiw@xidian.edu.cn (Wei Wei); mseoj@nus.edu.sg (Jianyong Ouyang); jjingchang@xidian.edu.cn (Jingjing Chang)

Text S1 Modeling of heterodimensional structure modulated aerogel shape experimentally and theoretically

During the freezing process of an aqueous dispersion, the water molecules undergo a phenomenon wherein they establish hydrogen bonds with one another across a broad spectrum. Consequently, the volume of these water molecules expands, resulting in the formation of loosely arranged crystals. Then the AWBps and MC are squeezed into the gaps of the randomly grown ice crystals, leading to the formation of a continuous AWBps/MC hybrid phase under the action of hydrogen bonding [S1]. Following this, in the process of desiccation, the ice crystals present in the uppermost layer of the frozen entity undergo heat absorption and sublimation. Consequently, the surface layer undergoes a transition from rigidity to viscosity, primarily due to the high concentration of cellulose. This transition, to some extent, restricts the release of water vapor from the lower layers and consequently decelerates the overall rate of sublimation. As a result, the heat absorption of the ice crystal sublimation is relatively reduced, and a cascade of heat cannot be conducted by the network of $AgBr_xCl_{1-x}$ clusters, owning lower thermal conductivity [S2]. Finally, the aggregate temperature of the solid entity experiences an increase. At the eutectic point, local melting takes place, resulting in the conversion of solid frozen water into its liquid state [S3].

Typically, the hydrostatic pressure due to capillary forces arising from the surface tension of melting water can be expressed as [S4]:

$$p_r = \frac{2\gamma \cos\theta}{r} \quad \text{(S1.1)}$$

where p_r is the capillary stress, γ is the surface tension of the liquid, θ is the contact angle of the liquid with the pore wall, and r is the radius of the pore. Herein, the aerogel pore sizes are in the micrometer range, it can be calculated from Eqn. (S1.1) that the skeletons would experience oblique pulling stresses of ~10 KPa as the liquids in the pores are evaporated. Additionally, the presence of large nanoparticles will exert additional force on the delicate and fragile skeletal structure primarily composed of short AgNWs and MC (Fig. S6). Constant compression of the AWBps/MC gel as water evaporates causes the gel to shrink and become dense, preventing the creation of low-density aerogel [S4, 5]. Additionally, when the pore radiuses on the two sides of a common wall are not equal, Eqn (1.1) dictates that force would destroy the structure of the AWBps/MC walls, eventually turning into an ultrathin and dense AWBps/MC aerogel film (Fig. 2a) [S5, 6].

While the solvent continues to sublimate and the gel as a whole does not receive any additional heat when the thermal conductivity channel (1D AgNWs) is widened and the thermal conductivity barriers (0D $AgBr_xCl_{1-x}$) are lowered (Fig. S7). In a solid state, capillary force is minimal, allowing for the stable formation of interlayer supporting filaments in aerogel foam (Figs. S8, S9 [S4, 7]. In contrast, very low concentrations of AWBps (meaning small amounts of $AgBr_xCl_{1-x}$) can also form an aerogel foam, whereas increasing the concentration of $AgBr_xCl_{1-x}$ will form collapsed foam (Fig. S10a, b). This also depicts that $AgBr_xCl_{1-x}$ particles are conducive to the formation of dense aerogel film, rather than low-density foam.

To verify this theory, we created aerogels with 500 nm particles and 50 nm particles to show how the presence of a large nanoparticle force influences aerogel formation. Clearly, the results confirm the external force of the large particles on the wall causes it to densify rather than porosity (Fig. S10c-f). Likewise, when a small amount of AgNWs is added to promote thermal conductivity, the resulting aerogel edges dissipate heat better than the central area due to the proximity of the edge to the mold. The end product is a structure of foam on the outside and film on the inside (Fig. S11).

Further, we analyze the mechanism of the heterodimensional structure modulated aerogel shape by percolation theory. Percolation theory for the above skeleton comes in three basic varieties: bond (including nanofillers of AWBPs and cellulose), site (3D space lattice), and continuum (aerogel), which help to standardize and analyze strongly disordered and random geometric nanomaterials based AWBps aerogel structures [S8]. Geometrically, 1D nanofillers (AgNWs and cellulose) are considered to be the most suitable fillers as they can easily form the percolation network structure [S9], also known as matrix, in composites. Yet, the probability of 0D AgNPs and $AgBr_xCl_{1-x}$ particles with low aspect ratios occupying the unit space lattice is relatively low, which means hinders the formation of a continuous skeleton compared with 1D fillers, therefore named impurity. Notably, the above theory suggests that AWBps (impurities dominated) alone may have trouble forming complete, dense aerogel. In addition, with more matrix filler in the unit spatial medium, more connections can be formed in the aerogel thickness direction, resulting in a “foam” aerogel structure with a significant thickness at the macro level, rather than “film” aerogel.

Overall, the concept of percolation theory provides a fundamental framework for understanding how the dimensionality of fillers (1D and 0D) influences the structural morphology of nanomaterial-based aerogels. The percolation threshold is defined as the critical concentration of filler materials at which the network transitions from an isolated, disconnected state to a continuous, percolative network. This threshold plays a pivotal role in determining whether the aerogel adopts a foam or film structure.

In the case of foam-type aerogels, where a robust, three-dimensional network is desired, the 1D fillers (such as silver nanowires) must reach a specific percolation threshold to enable the formation of thermally conductive, continuous pathways throughout the aerogel. As the concentration of 1D fillers increases and surpasses this threshold, the system transitions to a percolative regime, resulting in a foam structure with a well-connected, three-dimensional matrix. In contrast, when the filler composition favors 0D fillers (e.g., nanoparticles), the network fails to form long-range connectivity due to the limited geometrical aspect ratio of the fillers. These 0D fillers act as "impurities", disrupting the formation of a percolative network and thereby favoring a film-like structure that is less interconnected and more two-dimensional in the height direction.

The interplay between the 1D and 0D filler ratio is critical in governing the percolation threshold, which directly influences whether the aerogel forms a foam or a film structure. This relationship has profound implications for the resulting physical properties, such as mechanical integrity and EM behavior, as the percolative nature of the filler network dictates the aerogel's overall functionality and performance.

Experimentally, 0D nanofillers dominated film (Fig. 3a), 1D-0D nanofillers balanced "foam & film" hybrid (Fig. S11), MC dominated foam (Fig. S10b, e) and AgNWs dominated foam (See next section, Fig. 4a) were prepared successively according to the nanofiller proportion, and the morphology of these foams could prove the above conjecture. Therefore, the heterodimensional nanofillers can change the ratio of "matrix-impurity" to form disparate "film/foam" aerogel, thereby modulating the corresponding EM response.

Text S2 Design of multiscale cellulose matrix

First of all, we designed a multiscale micro/nanofiber network matrix for AWBps aerogel by the physical entanglement and high-density hydrogen bonding networks between the nanofibers (cellulose nanofibrils, CNF) and microfiber (bacterial cellulose, BC) (Fig. S12). The cellulose-based matrix not only boosts interfacial polarization due to the severe mismatch of conductivity in the interfaces, but also endows these composites with exceptional comprehensive mechanical properties, superior impact resistance and toughness [S6, 10]. Multiscale interface engineering endows cellulose-based structural materials with both the long-range physical connection of micro-materials (Fig. S13a, b) and the dense structure of nano-materials (Fig. S13c). As shown in Fig. S13d, the multiscale materials consist of microfibers with larger diameters that are intricately woven together both longitudinally and transversely, resulting in the formation of a robust 3D network skeleton.

Moreover, when observed using SEM, the nanofibers exhibit a notable interconnected structure due to the existence of a visible network of microfibers. This interconnection is facilitated by the nanofibers attaching to the microfibers through strong hydrogen bonds and interactions involving positive and negative charges [S11, 12]. The process involves the continuous intertwining of microfibers and nanofibers to create a compact dual network structure (Fig. S13d). The resulting material exhibits a density that falls between that of materials made solely from microfibers and those made solely from nanofibers. As a result, structural materials based on cellulose at multiple scales exhibit concurrent high strength and toughness.

Text S3 Analysis of microwave loss mechanism about AWBps/AgNWs aerogel foam

The dissipation of incident EM waves is mainly in the form of magnetic and dielectric losses,

which are described by magnetic permeability (μ', μ'') and dielectric constant (ϵ', ϵ''), respectively. The real parts ϵ' and μ' stand for the storage abilities of the electric and magnetic energies, respectively, whereas the imaginary parts ϵ'' and μ'' are related to the dissipation abilities of the electrical and magnetic energies carried by the incident EM waves, respectively. Fig. S22 shows the relative complex permittivity ($\epsilon_r = \epsilon' - j\epsilon''$) of non-magnetic AWBPs/AgNWs aerogel foam (S1, S2 and S3). And dielectric loss tangent (tan $\delta = \epsilon''/\epsilon'$) is usually used to evaluate the dielectric loss capacity of electromagnetic wave absorbers (Fig. 4h).

For low AgNWs aerogel, the dielectric constant and dielectric loss tangent tended to improve with the enhancement of AgNWs/AWBps ratio (Fig. S22). In order to analyze the dielectric loss mechanism of AWBps/AgNWs aerogel foam, the Cole-Cole curve was drawn according to the Debye theory, as shown in Fig. S22. The Cole-Cole curve of EG had long straight lines and small semicircles. This may be attributed to the conduction loss and polarization loss. The mesh skeleton of nanosilvers, especially AgNWs had good electrical conductivity, which was conducive to the generation of conduction loss [S13, 14]. Lattice defects in $AgBr_xCl_{1-x}$ were conducive to triggering the dipole polarization under the action of EM wave, resulting in polarization loss [S15]. Compared with nanosilvers-only foam, the number of semicircles in $AgBr_xCl_{1-x}$ based foam enhanced with distorted shape, displaying the mechanism of multiple polarization. The hinderance of charge flow in AWBps can be attributed to the presence of a heterogeneous interface, which consequently leads to an uneven distribution of electrons at the interface and facilitates interface polarization [S16]. In addition, cation defects in $AgBr_xCl_{1-x}$ components were favorable for dipole polarization.

In the case of S1, the observed disparity in permittivity between the two sides can be attributed to the presence of a conductive gradient. Simultaneously, the lower surface undergoes densification as a result of capillary forces, resulting in a scarcity of macroscopic pores. In other words, the bottom dielectric constant of this MC dominated foam is similar to that of the corresponding film, and the overall is higher. In the case of S2, the incorporation of a minor quantity of AgNWs facilitates the augmentation of the overall porosity, while a substantial presence of air pores at the bottom significantly decreases the dielectric constant [S17]. Furthermore, it is evident that the dielectric loss tangent of both materials exhibits a higher level of dielectric loss when subjected to top incidence mode across a majority of frequency bands. Additionally, S1 and S2 exhibit several broad formants within the frequency range of 14.0-17.0 GHz. This observation suggests the existence of pronounced polarization relaxation caused by the interface polarization effect of internal nanofillers [S16]. Further, after the introduction of more AgNWs, the dielectric constant and dielectric loss tangent increased significantly. S3 displayed a high dielectric constant and dielectric loss tangent (Figs. 4h and S22). Although the dielectric loss is greatly increased, the impedance mismatch results in a significant decrease in the MA performance (Fig. 4f) [S18, 19].

Text S4 Microwave absorption performance of AgNWs aerogel foam

AgNWs are typical one-dimensional materials with good electrical conductivity [S20]. It is widely used in EMI shielding materials with ultrahigh conductivity. Therefore, the large impedance mismatch makes it unsuitable for absorbing materials. However, the improvement of impedance matching by the multiscale pores of aerogels shown in Fig. S31, makes it have the function of MA under low fillers (-16.5 dB, 4.8 GHz@0.25 wt% and -23.9 dB, 1.2GHz@0.3 wt%, Figs. S32a_1, b_1). Nevertheless, it is obviously difficult to achieve compatibility between high EAB and low RL_{min}. This also explains the necessity of introducing $AgBr_xCl_{1-x}$ clusters,

even when it is not conducive to the formation of MA porous structures. Specifically, according to the Maxwell-Wagner-Sillars polarization principle, the porous structure and the numerous interfaces, synergizing with the large conductivity mismatch among nanosilver, $AgBr_{1-x}Cl_x$ and cellulose matrices, amplify charge storage capacity and enhance interfacial polarization [S21]. Apparently, the interfacial richness of silver nanowire aerogel is far less than that of by-product aerogel. As the filling amount of AgNWs increases, the Cole-Cole curve obviously decreases semicircular and tends to a straight line, which means that the conductive loss gradually increases and the polarization loss gradually decreases (Fig. S32a_2-c_2) [S13, S22].

When there are too many silver nanowires of aerogel, tan δ shows the increment of dielectric loss (Fig. S34). It is obvious that the impedance mismatch is severely aggravated due to ultrahigh conductivity shown in Fig. S33, and the absorption performance is poor (Fig. S32c_1).

In addition to the aforementioned performance of AgNWs aerogel, we have compiled the characteristics of AgNPs, AWBps, and AgNWs, as presented in Table S7.

Text S5 Mechanics of individual nanowires with multiphysics induced failure

During the life cycle of the AWBps EM function aerogel, it is obvious that it will be subjected to additional external forces (bend or twist) and the high-frequency EM waves it is protected against. At the same time, the crushing at the beginning of the final recovery and the external force of the stirring rotor will also cause mechanical damage to it. To specifically address the effects of deterioration on nanosilver (especially AgNWs), we categorize the influences into three key types: Cracks, Fragmentation, and Junctions. (i) Cracks refer to the presence of visible fissures or surface breaks in the AgNWs, where the wire has not completely broken apart but shows signs of structural compromise. These cracks can significantly weaken the electrical conductivity by disrupting the continuous path for electron flow while still maintaining partial connectivity along the wire. (ii) Fragmentation refers to the complete breakage of the AgNWs into smaller, disconnected pieces. This type of deterioration leads to a total loss of continuity in the conducting network, causing significant disruptions in electrical conduction. Fragmented nanowires no longer contribute effectively to the network, lowering conductivity and potentially increasing resistance. (iii) Junctions are formed when two or more AgNWs merge, either through physical contact or fusion during the recycling process. These junctions may create additional conductive pathways, but if poorly formed, they could result in irregularities that disrupt current flow or introduce weak points in the network.

Furthermore, the effect on the AWBps conducting network is as follows. (i) Cracks: Although cracks do not fully break the nanowires, they still disrupt the electrical continuity of the network, leading to localized increases in resistance. These cracked areas can act as weak points that are susceptible to further damage, especially under stress or over time, ultimately impairing the overall performance of the nanowire network. (ii) Fragmentation leads to a complete breakdown of the conductive network, with disconnected nanowire fragments no longer contributing to the conduction process. This results in a substantial decrease in overall conductivity and a higher resistance within the material. The aerogel's ability to perform functions like EMI shielding or electrical conduction is significantly reduced. (iii) The formation of junctions can have both positive and negative impacts. Well-formed junctions can enhance conductivity by providing additional pathways for electron flow, stabilizing the network. However, poorly formed junctions can cause local hotspots or discontinuities in the

network, potentially increasing electrical resistance and reducing the efficiency of the silver nanowire network.

Though AWBPs aerogel shows little variation in conductivity and EM function over many mechanical force, individual nanowires are shown to undergo brittle failure. The observed behavior can be attributed to dislocation starvation, a phenomenon characterized by dislocation starvation in nanoscale crystalline materials. This dislocation starvation has been found to diminish the ductility of the material, as evidenced by the behavior observed in single-crystal nanowires [S23]. Besides, the term dislocations starvation was coined to describe the plastic deformation controlled by the nucleation of new dislocations, rather than by the motion of pre-existing dislocations and activity of the internal dislocation sources [S24]. The nucleation of new dislocations in structurally perfect single crystals requires much higher stresses than those needed for the activation of pre-existing dislocation sources. This leads to a lack of dislocations in the material, which affects the plastic deformation of the material.

Nevertheless, it has been observed that AgNWs commonly display twinning, leading to the formation of dislocations that are subsequently affected by twin boundaries. Nanowires with dimensions exceeding 40 nm exhibit indications of dislocation starvation, leading to brittle failure, such as bending [S25]. However, when the diameter of the wire is reduced, the formation of dislocations on the surface becomes more prominent. These dislocations experience hindered motion due to the presence of twin boundaries. This phenomenon, known as strain hardening, results in dislocation motion being directly proportional to the deformation applied to the wire. Consequently, plastic deformation is facilitated, leading to an enhancement in ductility (Fig. S35) [S26]. Because the twin boundary area is smaller in relation to the surface area, surface dislocations are more likely to pile up and cause brittle fracture at larger diameters [S27]. This behavior is also observed at junctions, where the high curvature of a nanowire encasing the junction causes cracking and failure [S28]. Multiple minor breakdowns will alter the original conduction path, worsening local conductivity and eventually having an impact on the entire system.

Electromigration, triggered by conduction currents induced by EM fields, may also lead to cracks or breaks in AgNWs. The prevalence of electromigration induced failure (electrical breakdown) over the Rayleigh instability (thermal breakdown) was observed [S23]. This was attributed to the high thermal conductivity of Ag. The tendency toward electromigration-induced failure can be captured as follows [S29]:

$$\frac{\sigma / Z^{*2}}{\kappa} \sim \frac{\left(8e^2 \Delta T\right)}{\Omega^2 \left(\Delta s\right)^2} \tag{S1.2}$$

Where σ is the electrical conductivity, Z^* is the effective charge, κ is the thermal conductivity, Ω is the atomic volume, and e is the elementary charge. Δs, ΔT are mechanical stress gradient, and temperature difference ($T_{max} - T_{min}$) along the wire, respectively. Notably, electromigration can weaken the AgNWs network, and severe electromigration can cause the rupture of the AgNWs.

Supplementary Figures and Tables

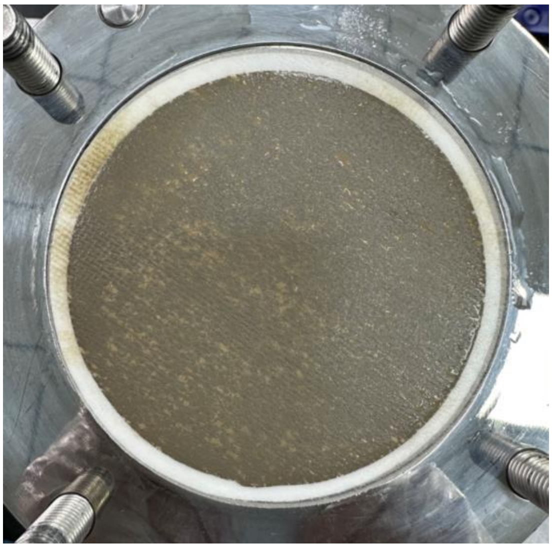

Fig. S1 Digital image of AgNWs on PTFE filter membrane after positive pressure filtration

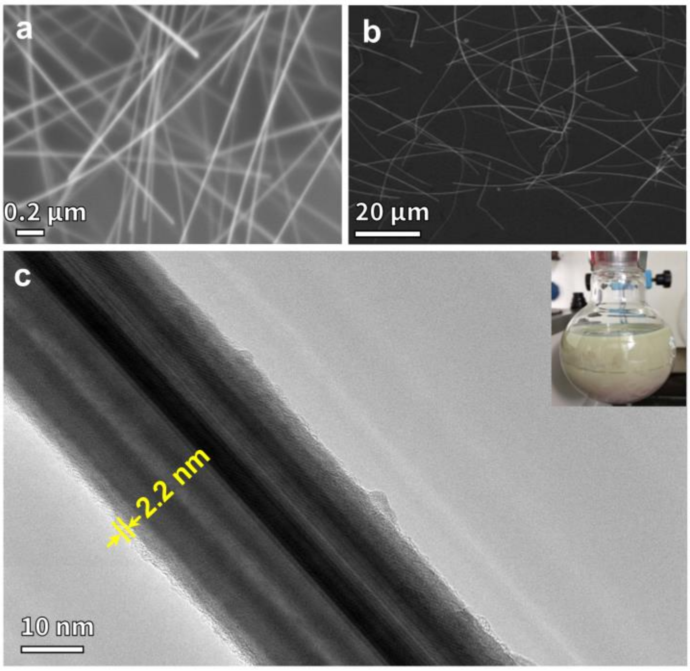

Fig. S2 SEM images of **(a)** low-magnification image shows the length of the AgNWs (~30 µm); **(b)** high-magnification image shows the diameter of the AgNWs (~30 nm), **(c)** TEM images of the as-prepared Ag NWs, which show polymer (PVP) on the wire surface indicated by yellow arrows (2.2 nm thick) (inset: the AgNW aqueous suspension)

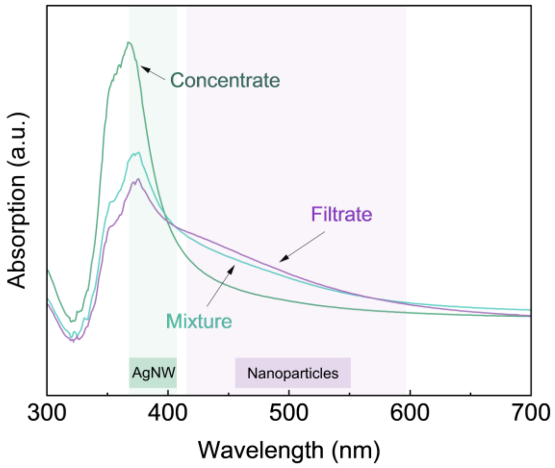

Fig. S3 UV/Vis spectra of a mixed dispersion of Ag nanowires, Ag nanoparticles, Ag nanorods and $AgBr_xCl_{1-x}$ before and after Positive-pressure filtration

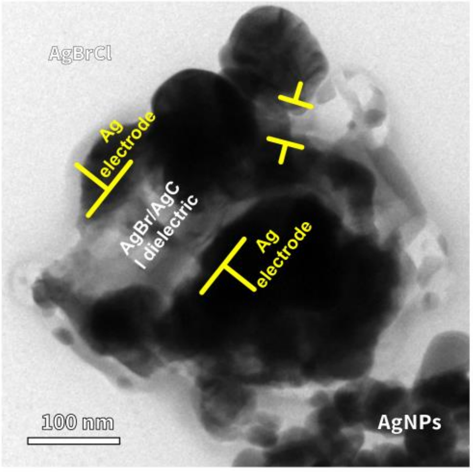

Fig. S4 TEM images of AgBr/AgCl@Ag cluster ($AgBr_xCl_{1-x}$) containing AgBr/AgCl microcapacitors. The dark-gray area is Ag. And light-gray area is AgBr/AgCl

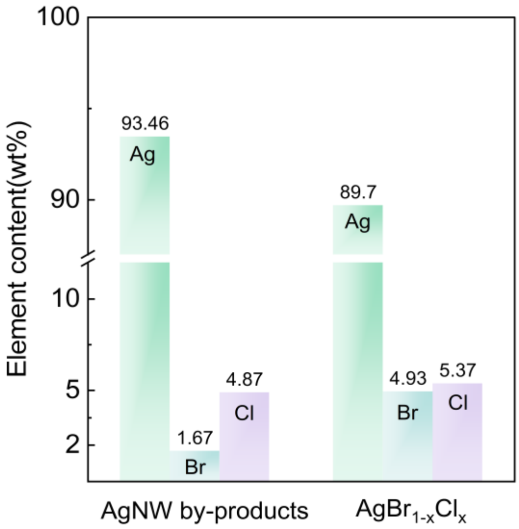

Fig. S5 Elemental contents of AgNW by-products (AWBps) and $AgBr_xCl_{1-x}$

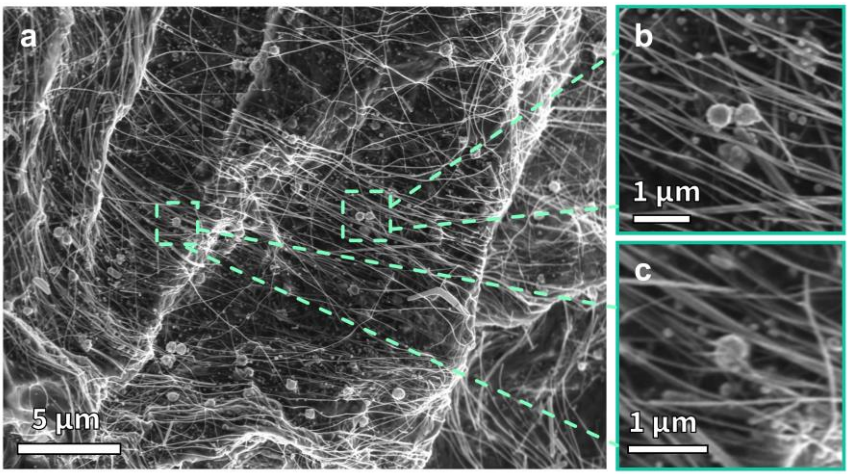

Fig. S6 SEM images of **(a-c)** interaction relation of AgNWs and $AgBr_xCl_{1-x}$

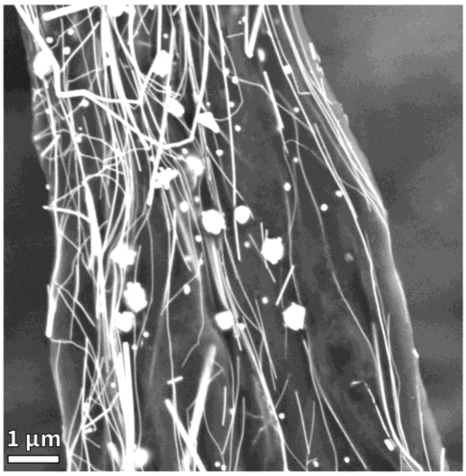

Fig. S7 SEM images of AWBps/AgNWs/MC connecting filament in the foam with continuous AgNWs constructed heat conduction paths

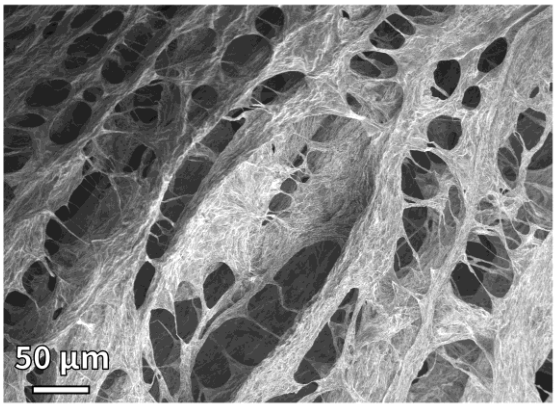

Fig. S8 SEM images of AWBps/AgNWs/MC aerogel with numerous connecting filaments

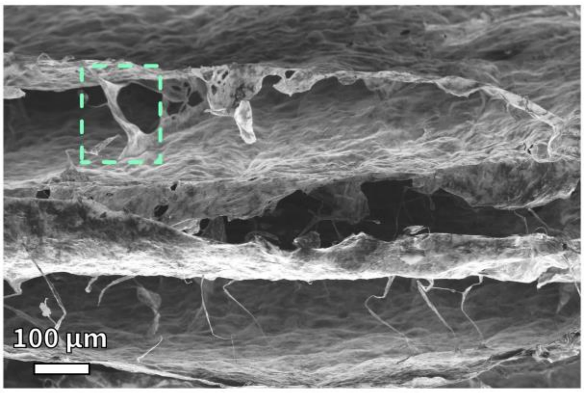

Fig. S9 SEM images of the nacre-like/bidirectional structure in the AWBps/AgNWs/MC aerogel

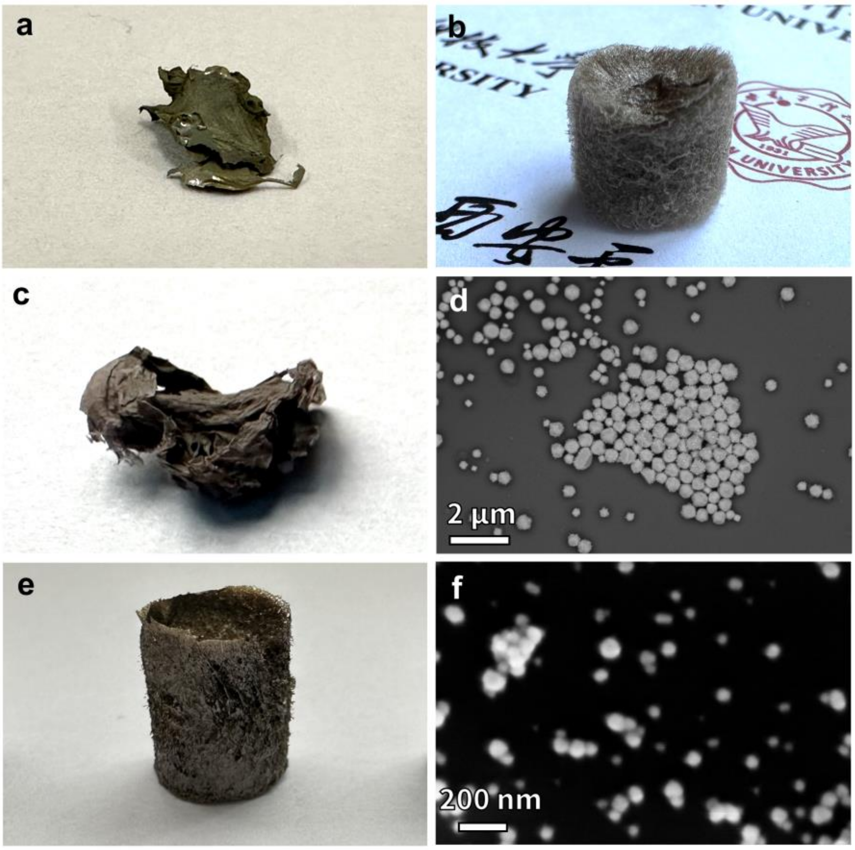

Fig. S10 Digital image of **(a)** high content AWBps aerogel (1 wt%); **(b)** low content AWBps aerogel (0.2 wt%); **(c)** Large AgNP aerogel; **(d)** SEM image of Large AgNPs, which average dimeter is 500 nm. **(e)** Digital image of tiny AgNP aerogel (1 wt%); **(f)** SEM image of tiny AgNPs, which average dimeter is 50 nm

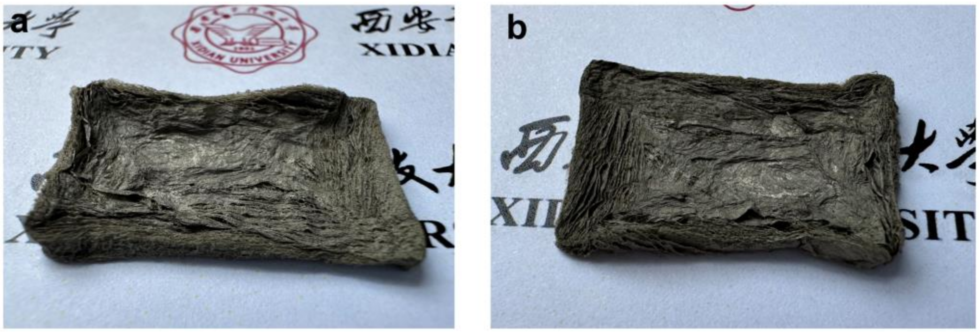

Fig. S11 Digital images of AWBps/AgNWs/MC aerogel foam, which possesses raised edges (like foam) and sunken interior (like film). **(a)** Front view; **(b)** Top view

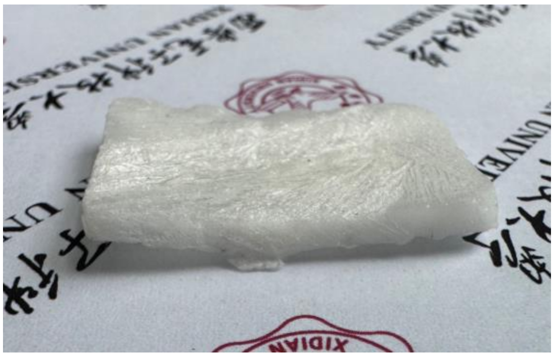

Fig. S12 Digital image of multiscale MC aerogel

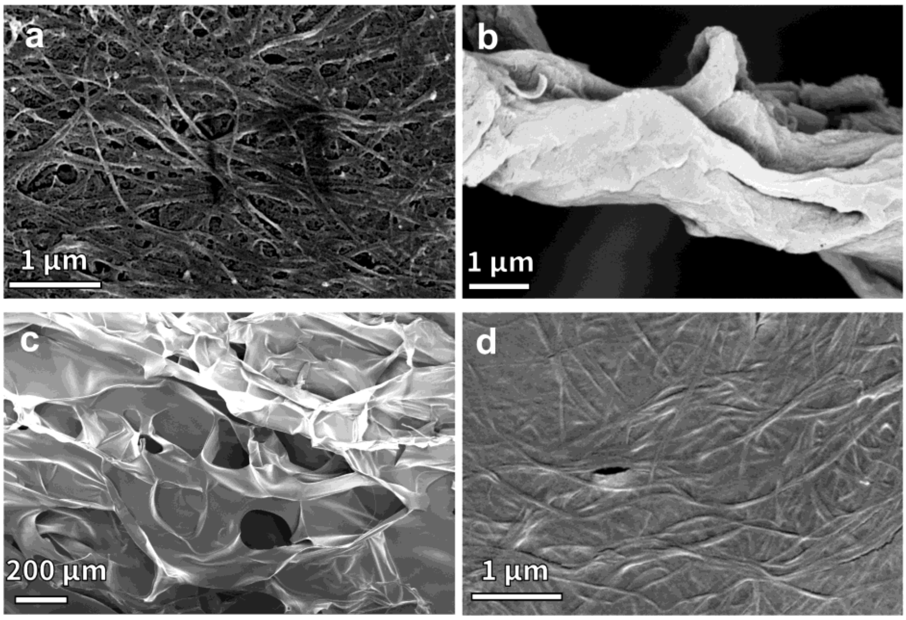

Fig. S13 SEM images of **(a)** Bacterial CNF film; **(b)** Bacterial CNF aerogel; **(c)** Nano CNF aerogel; **(d)** Bacterial CNF@Nano CNF film

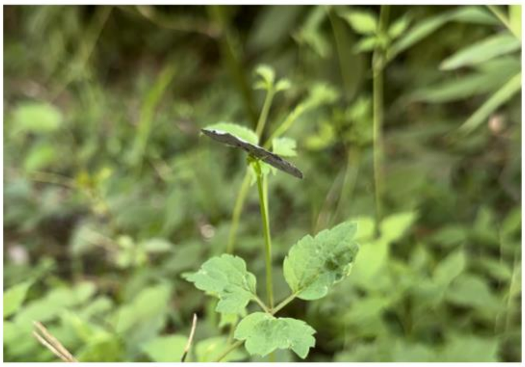

Fig. S14 Digital image of AWBps aerogel film (side view)

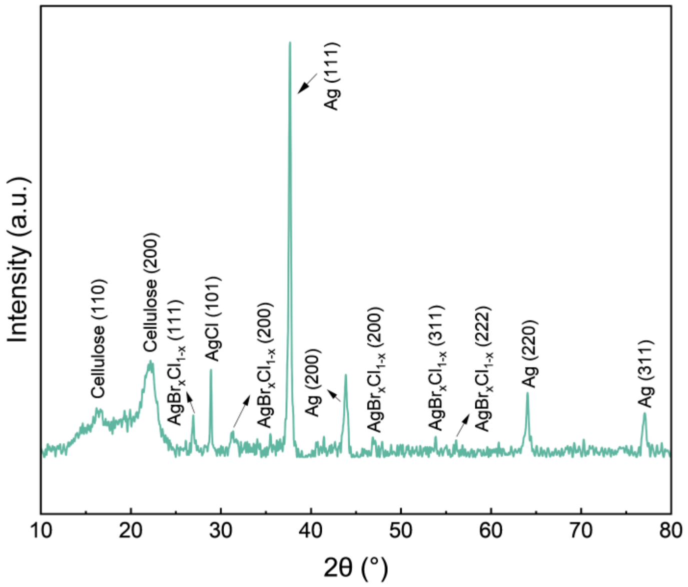

Fig. S15 XRD patterns of MC/AWBps aerogel

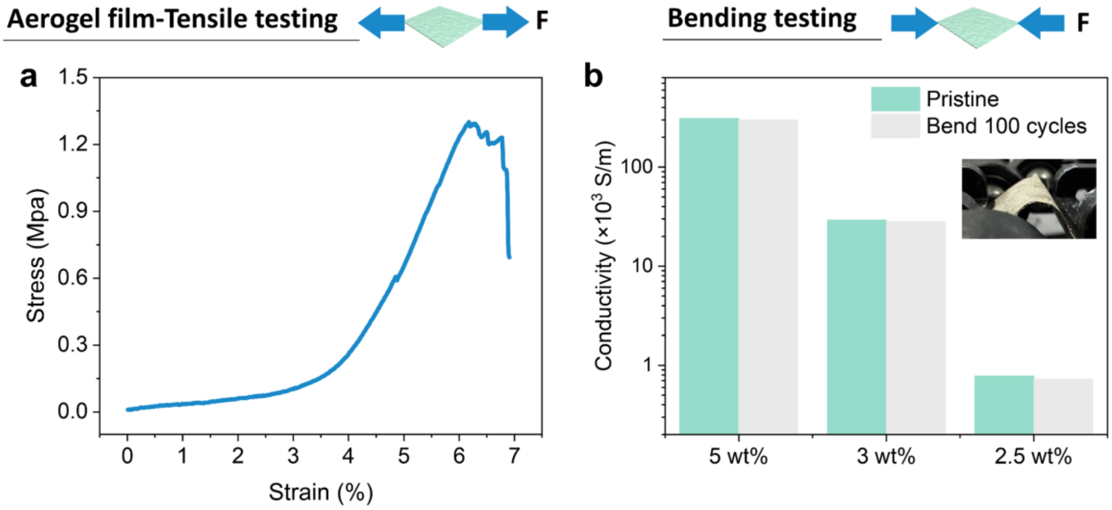

Fig. S16 Mechanical response of AWBps aerogel films. **a)** Stress-strain curve of AWBps based aerogel film in tensile test. **b)** Conductivity of AWBps based aerogel film before and after bend 100 times

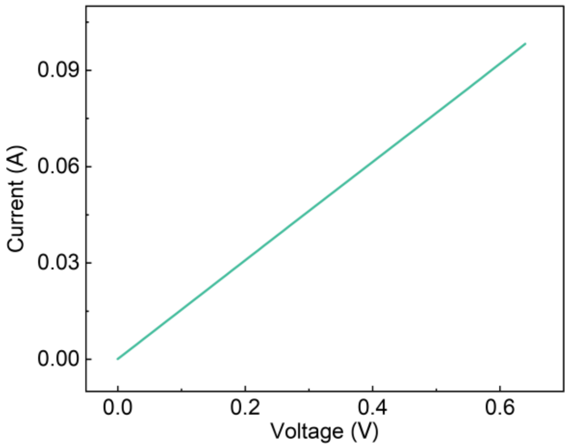

Fig. S17 A typical I−V curve of AWBps aerogel bulk at room temperature showing its pure resistance

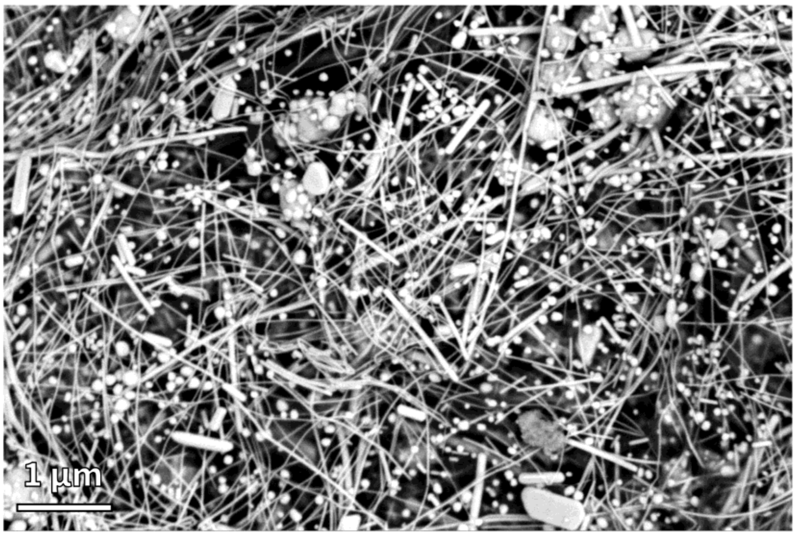

Fig. S18 SEM image of AWBps/MC aerogel film

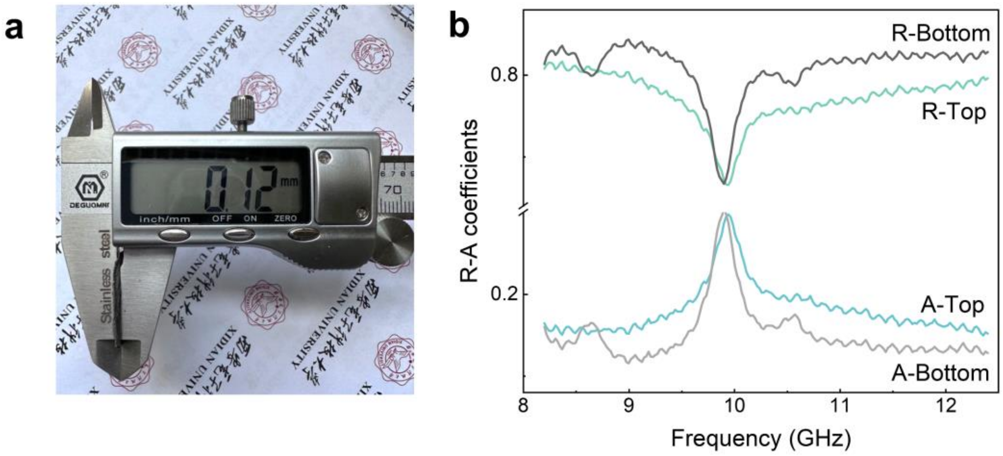

Fig. S19 (a) A cross-sectional digital image of the 2 wt% AWBps aerogel film (corresponding to Figs. 3c and 3g), revealing the specific thickness; **(b)** R-A coefficients of aerogel film when EM wave incidence from top or bottom surface

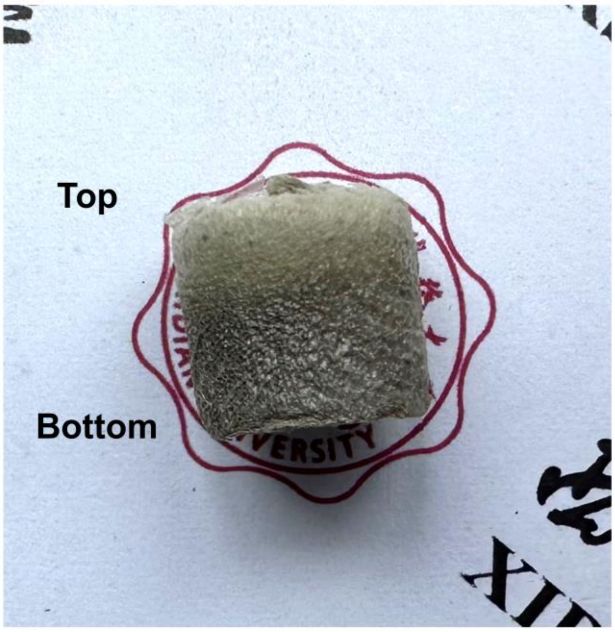

Fig. S20 Digital image of cylinder AWBps/AgNWs/MC aerogel foam with noticeable color difference from top to bottom. The darker the color, the more nanosilvers namely the lighter the color, the more MC

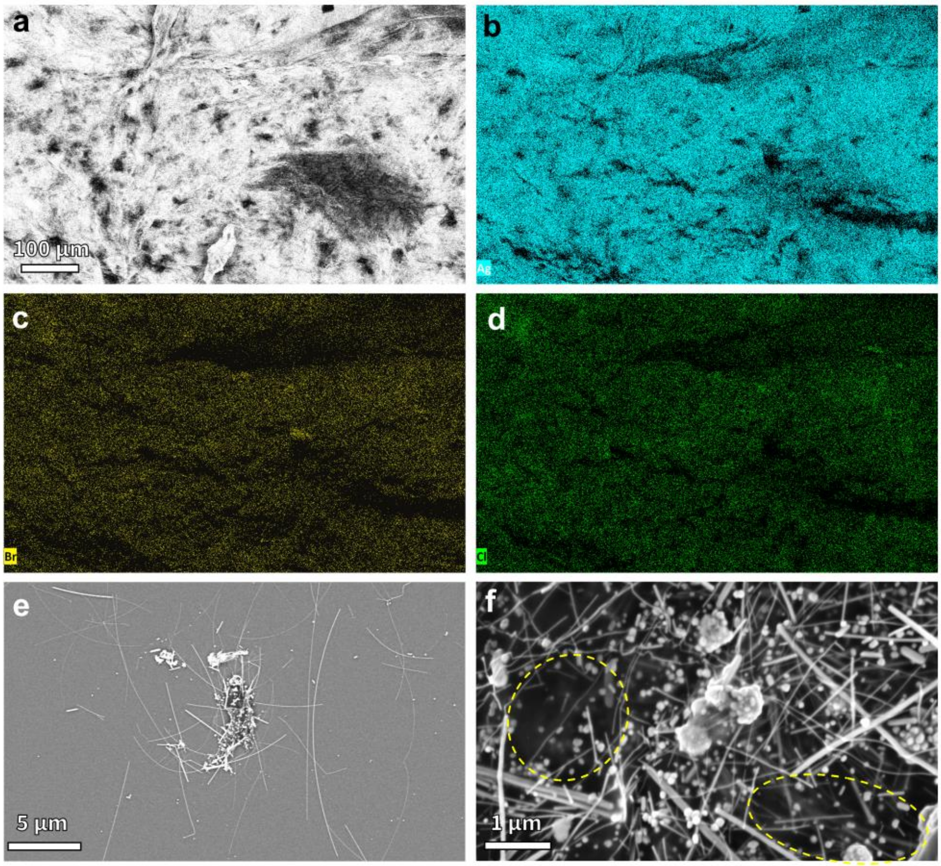

Fig. S21 (a-d) EDS maps of high content AWBps aerogel. **(e, f)** Large nanoparticle agglomeration

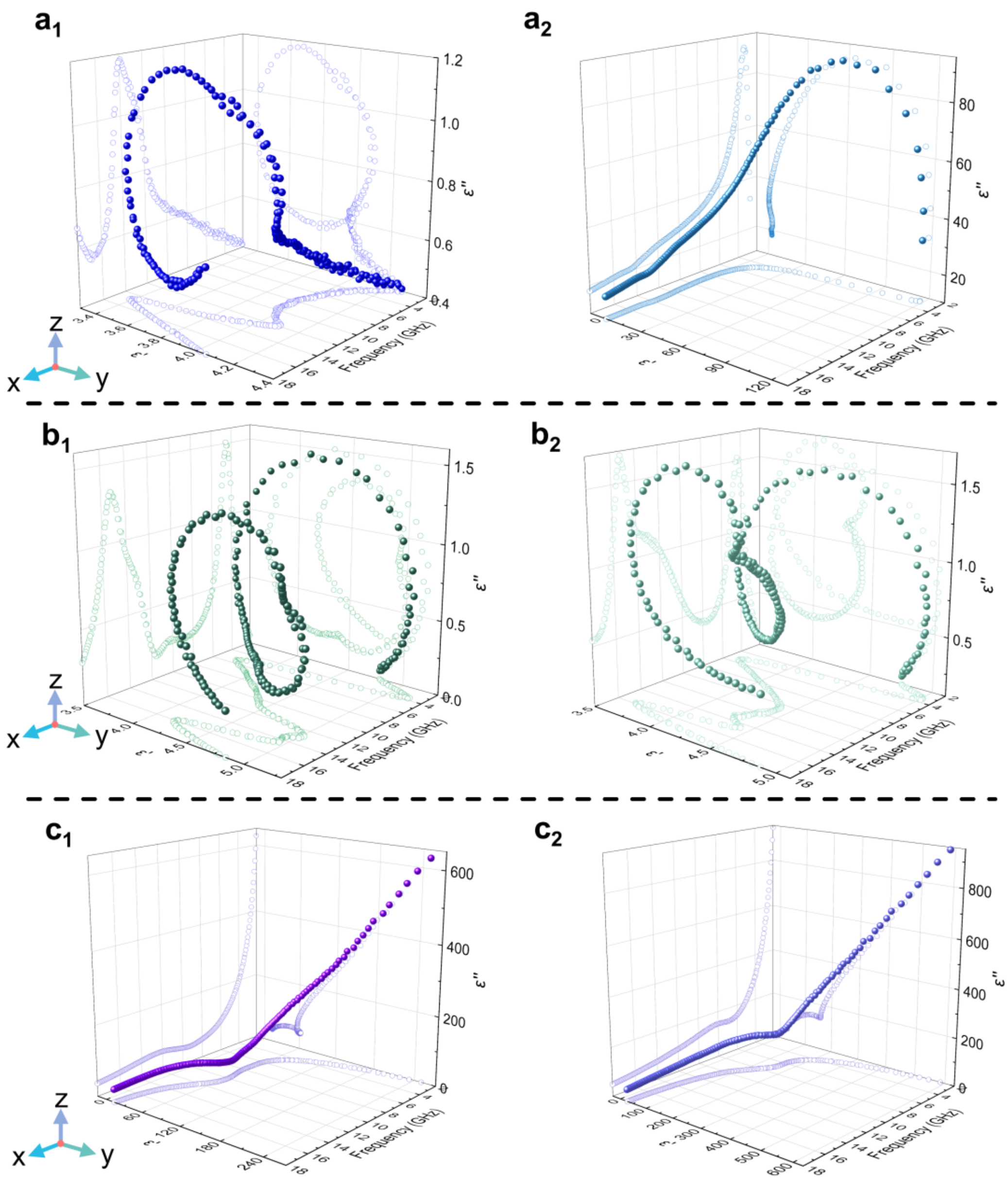

Fig. S22 EM parameter ϵ' (x-y plane), imaginary permittivity ϵ'' (x-z plane), and Debye relaxation curves (Cole–Cole semicircles, y-z plane) of AWBps/AgNWs/MC aerogel with different filler contents combinations: ABp-1 wt% **(a1)** Top incidence; **(a2)** Bottom incidence; ABp-1.6 wt%, AgNW-0.2 wt% **(b1)** Top incidence; **(b2)** Bottom incidence; AWBps-1.3 wt%, AgNW-0.4 wt% **(c1)** Top incidence; **(c2)** Bottom incidence

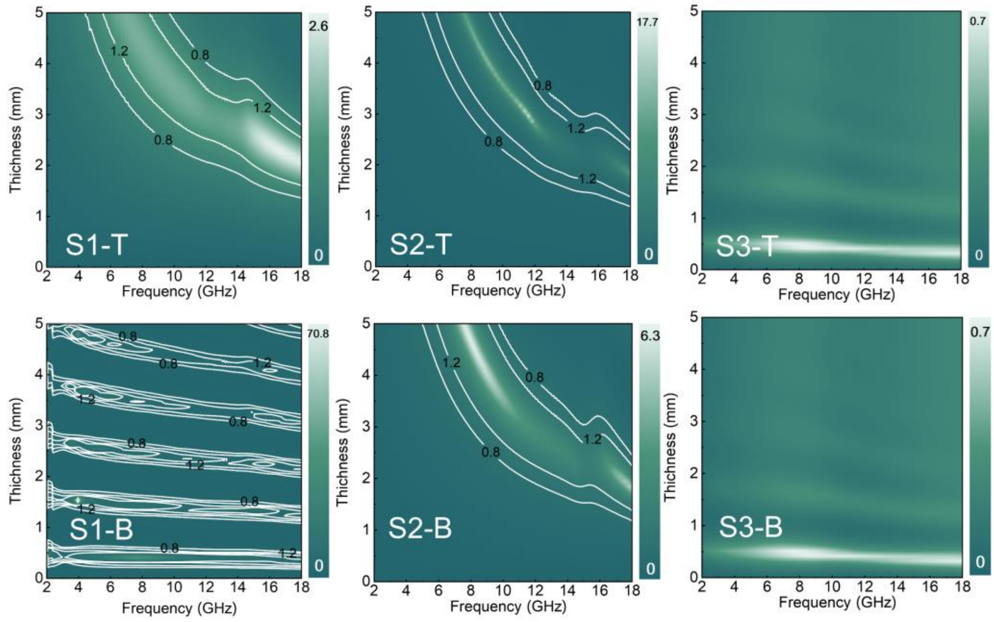

Fig. S23 Impedance match characteristic values of 1 wt% AWBps, 1.6 wt% AWBps-0.2 wt% AgNWs and 1.3 wt% AWBps-0.4 wt% AgNWs aerogel foam, containing top incidence and bottom incidence modes

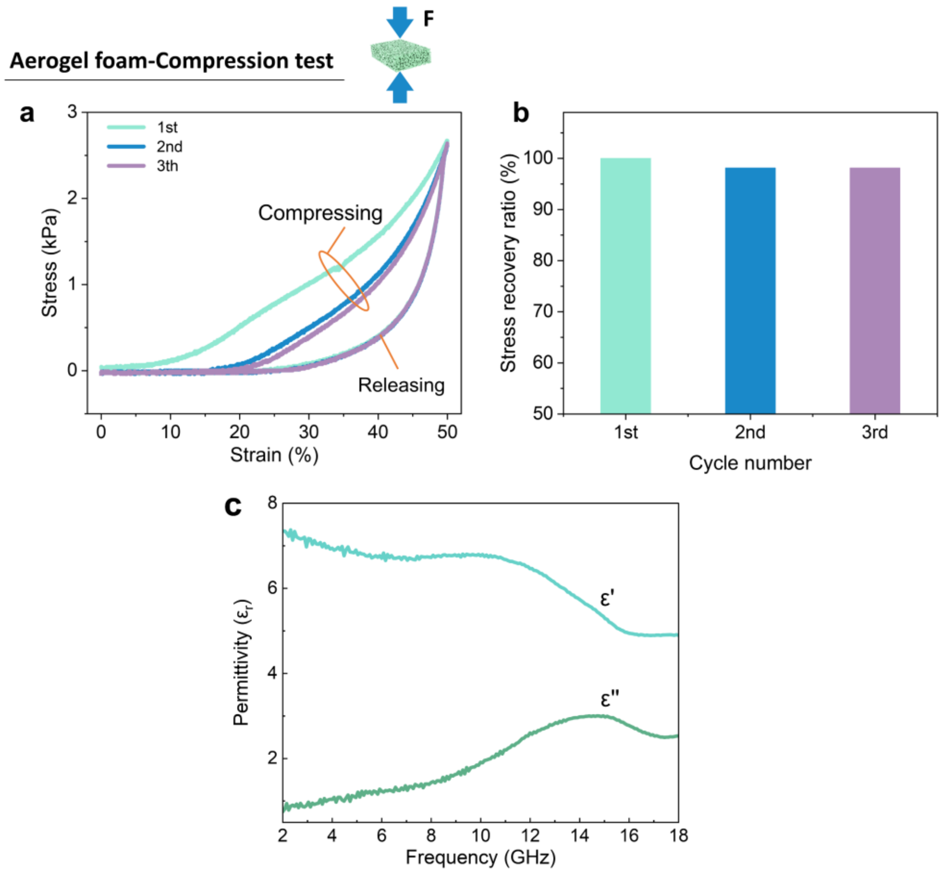

Fig. S24 (a) Stress–strain curves during compressing-releasing cycles at 50% strain, **(b)** The maximum compression strength recovery ratios for each cycle, and **(c)** Relative complex permittivity ($\varepsilon_r = \varepsilon' - j\varepsilon''$) of the AWBps/MC dual EM function aerogel foam

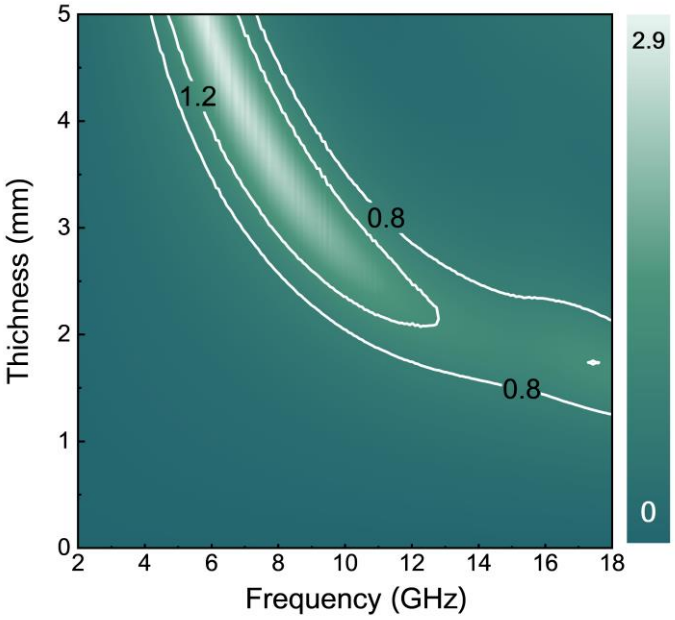

Fig. S25 Impedance match characteristic values of the AWBps/MC dual EM function aerogel foam

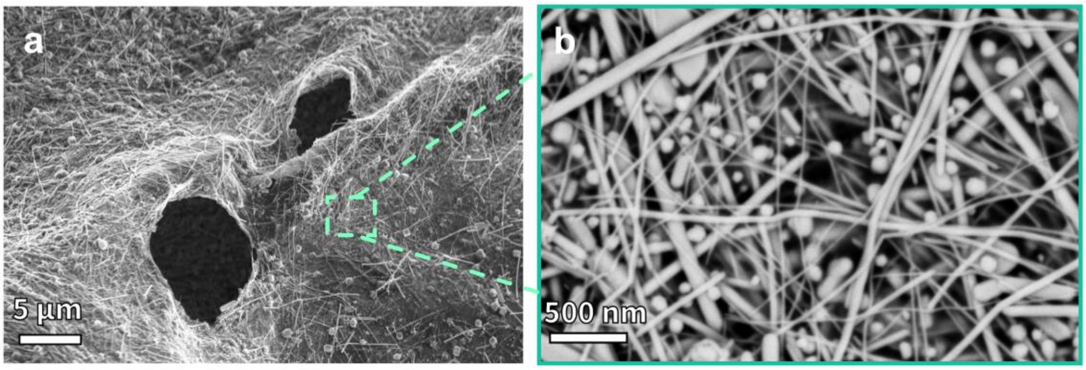

Fig. S26 SEM images of **(a, b)** AWBps/AgNWs/MC aerogel foam

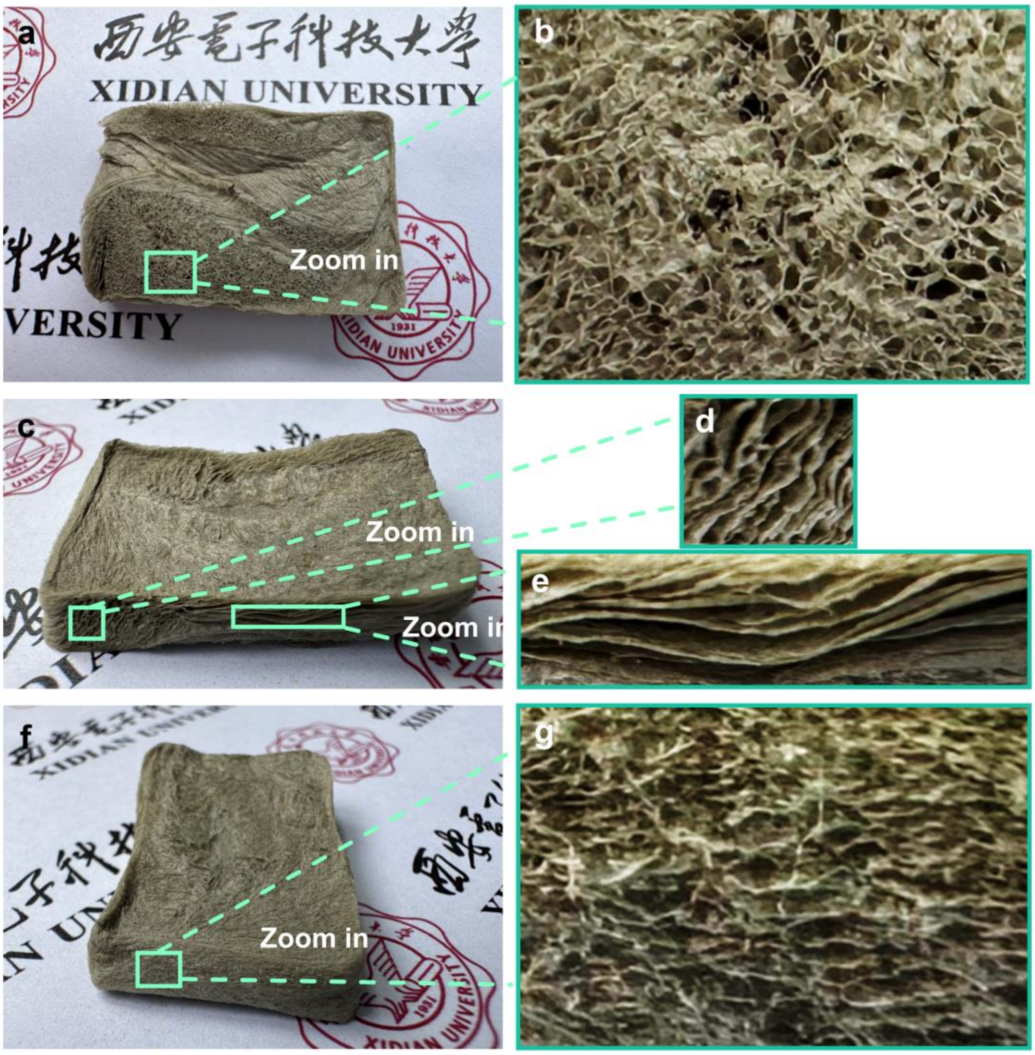

Fig. S27 Digital image of AWBps/MC aerogel. (a) Top view; **(b)** Magnified digital image of the sample shown in a, revealing the honeycomb-like/unidirectional holes; **(c)** Front view; **(d, e)** Magnified digital image of the sample shown in c, revealing the nacre-like/bidirectional holes. **(f)** Side view; **(g)** Magnified digital image of the sample shown in **f**, revealing the uniform/random holes. The contrast and brightness of the enlarged images are increased to enhance the display of various holes

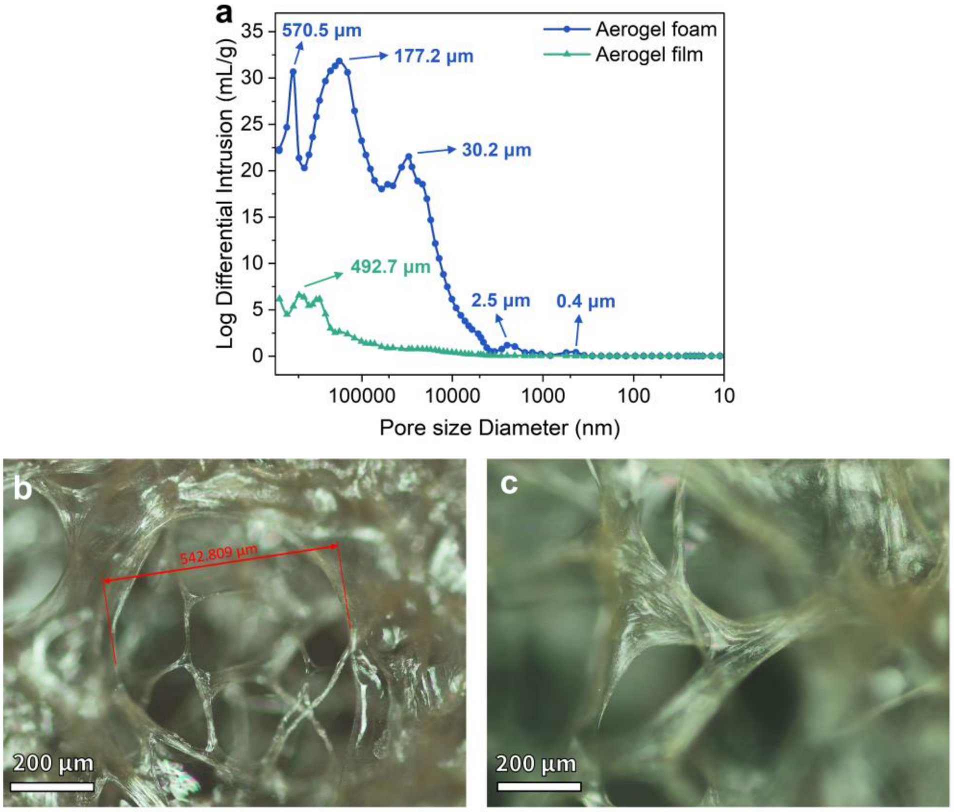

Fig. S28 (a) Pore size distributions of aerogel film and foam by mercury intrusion porosimetry (MIP). **(b)** Optical images of AWBps/MC aerogel. Inner Hole; **(c)** Interconnection area

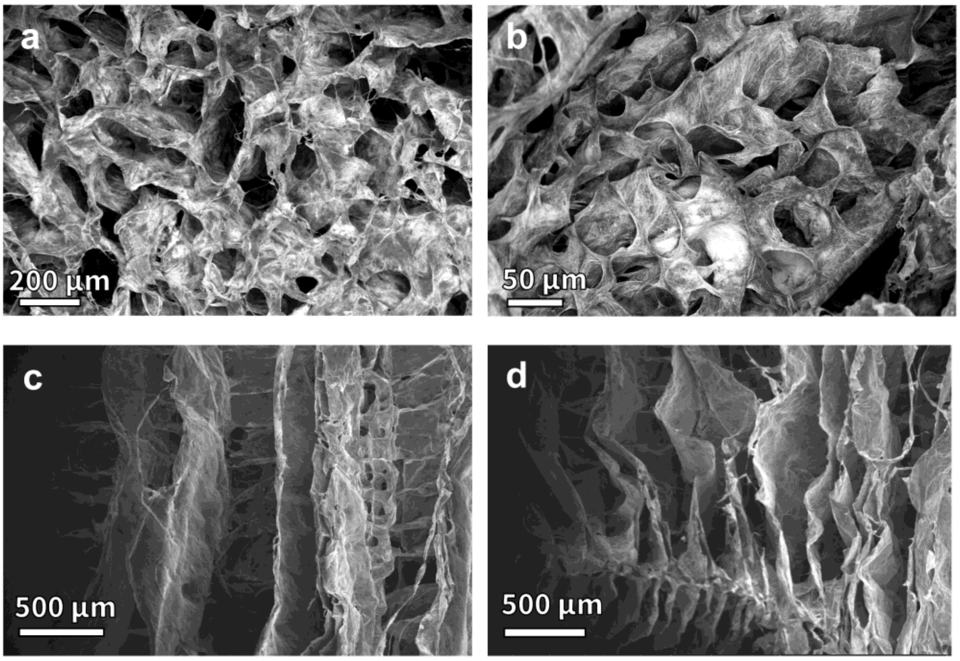

Fig. S29 SEM images of uniform/random holes with different sizes: **(a)** ~120 μm; **(b)** 70 μm and honeycomb-like/unidirectional holes with various channel sizes: **(c)** ~500 μm; **(d)** ~300 μm of AWBps/AgNWs/MC aerogel

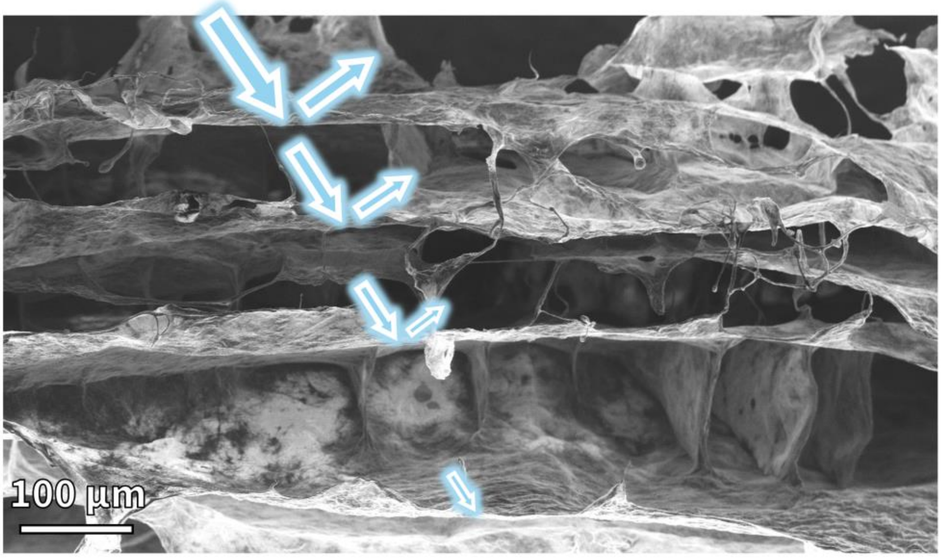

Fig. S30 SEM images of the nacre-like/bidirectional structure in the AWBps/AgNWs/MC aerogel

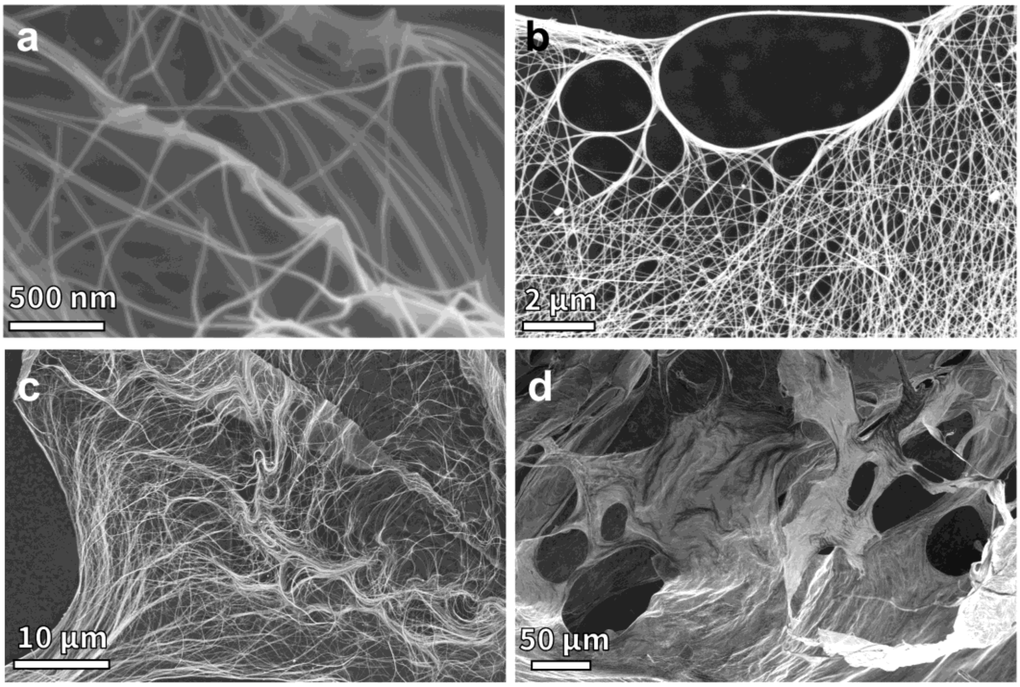

Fig. S31 SEM images of the various scale hole in the AgNWs aerogel: **(a-c)** nanoscale holes; **(d)** microscale holes

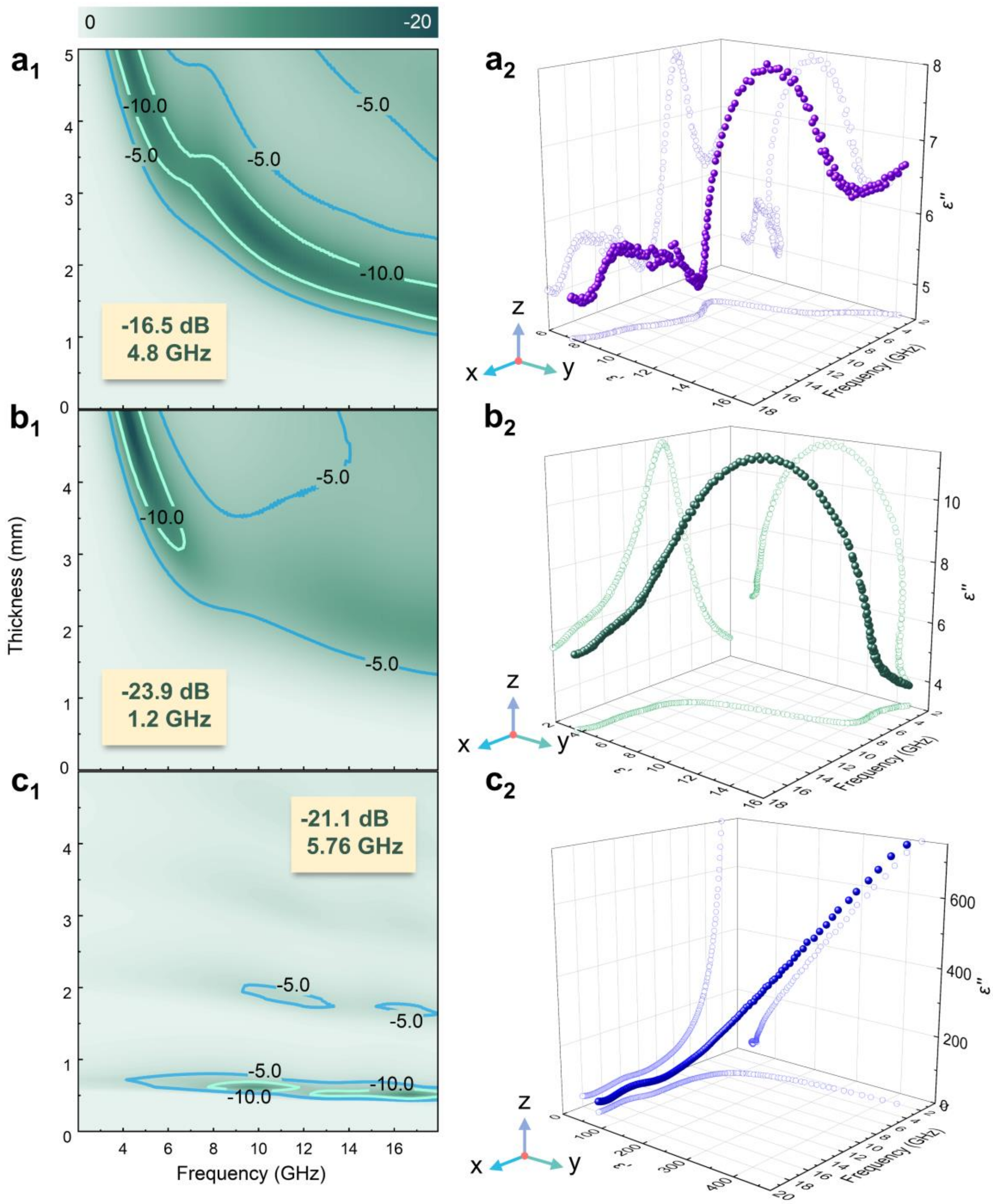

Fig. S32 Two-contour representations of the reflection loss (RL) values for AgNWs aerogel at different thicknesses: **(a_1)** 0.25 wt%; **(b_1)** 0.3 wt%; **(c_1)** 1 wt%. EM parameter ϵ' (*x-y* plane), imaginary permittivity ϵ'' (*x-z* plane), and Debye relaxation curves (Cole–Cole semicircles, *y-z* plane) of AgNW aerogel with different filler contents: **(a_2)** 0.25 wt%; **(b_2)** 0.3 wt%; **(c_2)** 1 wt%

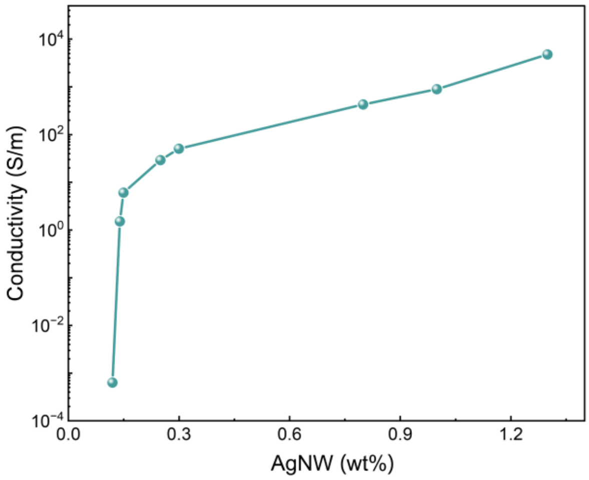

Fig. S33 Electrical conductivity of the cellulose/AgNWs aerogels with various AgNWs contents

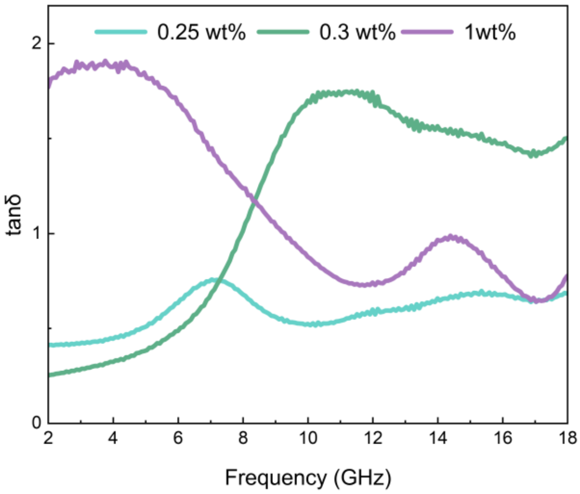

Fig. S34 Dielectric tangent loss of AgNWs aerogel foam

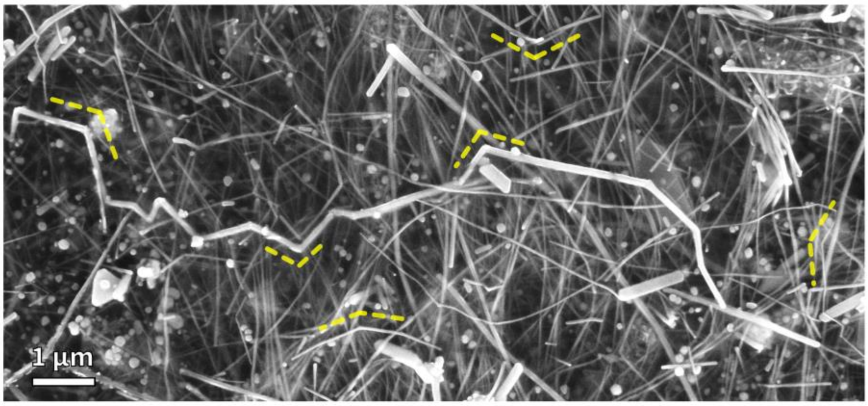

Fig. S35 SEM image of the failure (specific bend) of AWBps after secondary recycling

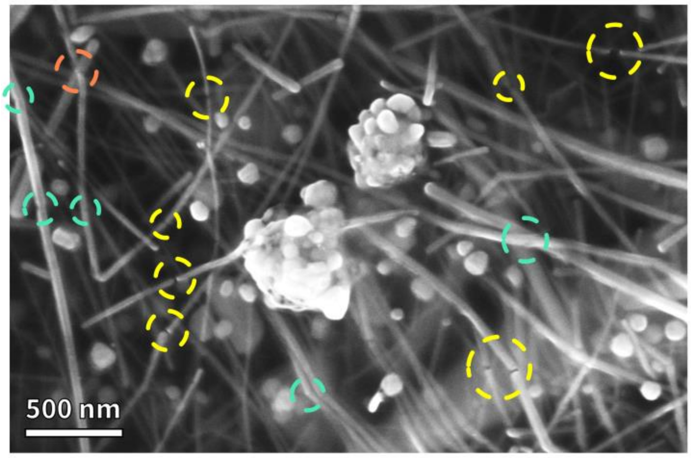

Fig. S36 SEM image of the failure (specific crack (green circled) and fragmentation (yellow circled)) of AWBps and its junctions (red circled) after secondary recycling

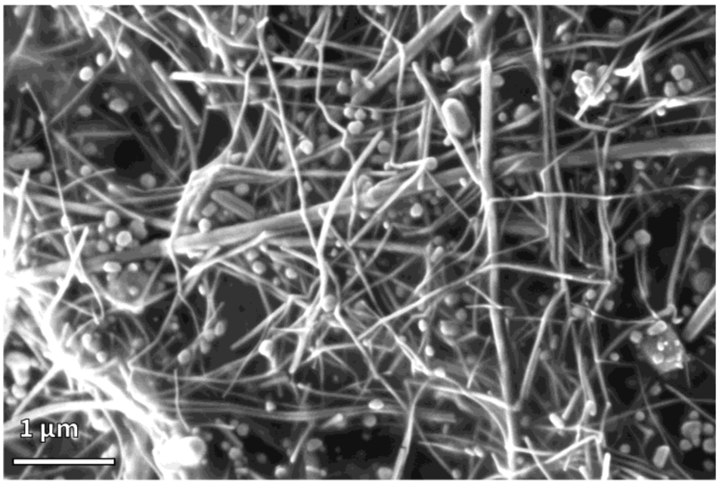

Fig. S37 SEM image of AWBps after secondary recycling

Table S1 The mechanical properties of various shielding films

Type	Filler & Matrix	Tensile strength [MPa]	Strain at breaking [%]	Refs.
Aerogel	MXene-CNFs	4.9±2.8	3.1±0.3	*ACS Appl. Mater. Interfaces (2023)[S30]*
Aerogel	CNF	~1.0	~7.3	*ACS Appl. Nano Mater. (2021)[S31]*
	50 wt% Silicone-coated MXene-CNF	~0.6	~4.2	
Hydrogel	PEDOT: PSS-ionic liguid	~1.3	19	*Adv. Mater. (2022)[S32]*
Aerogel	AWBps-BC-CNF film	1.3±0.1	7.55	**This Work**

Table S2 The mechanical properties of various shielding foams

Type	Filler & Matrix	Compression strength [kPa]	Compression strain [%]	Refs.
Aerogel	rGO-CNFs- glucose carbon	~4.2	70	*ACS Appl. Mater. Interfaces (2024)[S33]*
Aerogel	AgNWs-CNF	~5	\	*ACS Nano (2020) [S34]*
	75 wt% AgNWs-CNF	~2	\	
Aerogel	hexagonal boron nitride nanoribbons-orthogonally structured graphene	~3.9	40	*ACS Nano (2022)[S35]*
Aerogel	MXene	1.6	50	*Adv. Optical Mater. (2019)[S36]*
Aerogel	CuNWs-graphene	~3	50	*Small (2018)[S37]*
Aerogel	AWBps-BC-CNF foam	~2.6	50	**This Work**

Table S3 EMI shielding performance of various shielding films

Type	Filler	d (thickness) [mm]	Conductivity [S/m]	EMI SE [dB]	SE/d [dB/mm]	Refs.
Carbon-based shields	Graphene	1.12	2.1×10^3	106.9	95.4	*Adv. Mater. (2022)[S38]*
	Graphene	2	340	70.9	35.5	*ACS Nano. (2022)[S35]*
	Graphene	0.2	1.3×10^5	93	465.0	*Adv. Mater. (2020)[S39]*
	CNT	2	2.6×10^3	100	50.0	*Nano Lett. (2021)[S40]*
	CNT	0.101	8540	54	534.7	*ACS Nano. (2022)[S41]*
	CNT	2.3	44.6	50.5	22.0	*Adv. Funct. Mater. (2016)[S42]*
	CNT	2	516	40	20.0	*Adv. Funct. Mater. (2016)[S43]*
	CNT	0.568	230	57	100.4	*ACS Nano. (2022)[S44]*
	CNT/graphene	1.6	118	47.5	29.7	*Adv. Mater. (2017)[S45]*
	Carbon black	1.73	17.3	25.5	14.7	*Matter (2021)[S46]*

	Carbonized wood/ZIF-8※	0.14	5.8×10^3	46	328.6	*Adv. Funct. Mater. (2023)[S47]*
	Carbon Tube	1	300	47.2	47.2	*Nat. Commun. (2023)[S48]*
MXene-based shields	MXene	0.08	3×10^4	85	1062.5	*Adv. Mater. (2023)[S49]*
	MXene	1	1.28	28	28.0	*Angew. Chem. (2023)[S50]*
	MXene	2	1081	62	31.0	*Adv. Funct. Mater. (2019)[S51]*
	MXene/PPy	1.3	1×10^3	90	69.2	*Adv. Funct. Mater. (2019)[S52]*
	MXene/AgNW	0.12	1×10^4	54	450.0	*Adv. Funct. Mater. (2019)[S53]*
	MXene/AgNW	0.091	3.7×10^5	80	879.1	*ACS Nano. (2020)[S54]*
	MXene/AgNW	1	8×10^4	44.96	45.0	*ACS Nano. (2022)[S55]*
	MXene/graphene	3	120	51.4	17.1	*Adv. Funct. Mater. (2023)[S56]*
Other shields	LM	3	1.98×10^6	75	25.0	*Adv. Funct. Mater. (2022)[S57]*
	LM	1	1.1×10^6	81	81.0	*Adv. Mater. (2020)[S58]*
	PEDOT:PSS /MXene	0.12	>12	61	508.3	*Adv. Funct. Mater. (2023)[S59]*
Silver-based shields	AgNP	5	1.4×10^3	84	16.8	*Compos. Pt. A-Appl. Sci. Manuf. (2020)[S60]*
	AgNP	1.4	223.7	82.02	58.6	*Compos. Pt. B-Eng. (2022)[S61]*
	AgNP/ Modified-LM	0.08	2×10^5	38.5	481.3	*Adv. Funct. Mater. (2023)[S62]*
	AgNP/rGO/FeCo	0.4	3.2×10^4	72.5	181.3	*Compos. Pt. B-Eng. (2020)[S63]*
	AgNW	2	1×10^3	70.5	35.3	*ACS Nano. (2019)[S34]*
	AgNW	0.3	1.66×10^4	52	173.3	*Adv. Funct. Mater. (2021)[S64]*
	AgNW	0.66	2×10^3	55	83.3	*Angew. Chem. (2022)[S65]*
	**AgNW by-products**	**0.12**	**3.1×10^5**	**94.5**	**787.5**	**This Work**

※ZIF-8: Zeolitic imidazolate framework-8. Values of EMI SE indicate maximum value in measured range.

Table S4 EMI shielding performance of shields following the "waste-to-wealth" strategy

Type	"Waste" filler	Thickness [mm]	Conductivity [S/m]*	EMI SE [dB]	Treatment	Refs.
Biomass-based shields	LSW§	2	1943.6	62.66	Shear milling	*J. Mater. Chem. C (2021)[S66]*
	LSW/Ag	0.4	1.45×10^{4}	88.9	Shear milling	*ACS Appl. Mater. Interfaces (2020)[S67]*
	LSW/Ag	1.2	R: 2 Ω	62	Shear milling	*ACS Sustainable Chem. Eng. (2022)[S68]*
	LSW/PANI	2.5	11	50	Shear milling	*ACS Appl. Mater. Interfaces (2021)[S69]*
	LSW/PAN/ ZIF-67/Co@CNF	0.25	45	49	800°C carbonization Acid hydrolysis	*ACS Appl. Mater. Interfaces (2022)[S70]*
	LSW/PAN/AgNW	0.15	1×10^{5}	80	Acid hydrolysis	*J. Colloid Interf. Sci, (2022)[S71]*
	Waste cotton/Ni◎	1.4	$R_{\square}$: 0.84 Ω/□	107	900°C carbonization Alkali treatment	*Carbon (2023)[S72]*
	Waste carbon nonwoven-fiber\Al	8	80.64	80	500°C carbonization	*Chem. Eng. J. (2022)[S73]*
	Straw	1.7	\	57.7	1500°C carbonization	*ACS Sustainable Chem. Eng. (2019)[S74]*
	Pinecone	4	11	54.51	1000°C carbonization	*Polym. Composite. (2023)[S75]*
	Banana leaf	5	8.7	77.5	900°C carbonization	*Carbon (2023)[S76]*
	Carbon fibre waste	4.48	33.83	85	No extra treatment	*J. Clean Prod. (2021)[S77]*
	Waste tissue paper	4	123	40.8	900°C carbonization	*Chem. Eng. J. (2020)[S78]*
	Corn straw/ ZIF-67	4.91	$>1\times10^{4}$	45	800°C carbonization	*Nano Energy (2023)[S79]*
Others	Red mud/CNT	2	2400	83.4	Hazardous waste disposal	*J. Clean Prod. (2022)[S80]*
	Aluminum-plastic packaging/ Expanded graphite	2	47.7	106.4	Shear milling	*Compos. Pt. A-Appl. Sci. Manuf. (2021)[S81]*
Nanofiller-based shields	MXene sediment	0.51	663.34	45.3	No extra treatment	*ACS Appl. Mater. Interfaces (2022)[S82]*
	MXene sediment	7.5	1.7	91	No extra treatment	*ACS Nano (2022)[S83]*
	MXene/MXene sediment	0.43	7×10^{3}	78	No extra treatment	*Adv. Funct. Mater. (2023)[S84]*
	**AgNW by-products**	**0.12**	$\mathbf{3.1\times10^{5}}$	**94.5**	**No extra treatment**	**This Work**

§: LSW: Leather solid waste; *: Except for special marks, the conductive performance of the remaining materials is expressed by the conductivity [S/m]; ◎: Ku-band (12.4-18 GHz).

Table S5 Microwave absorption performance of various metal element containing absorbers

Type	Filler	Thickness [mm]	EAB [GHz]	Annealing	Refs.
Fe containing absorbers	Fe_3C	2.9	2.55	Yes	*Nat. Commun. (2024)[S85]*
	FeCo@C	4	2.58	Yes	*Adv. Funct. Mater. (2023)[S86]*
	$Fe(CO)_5/Fe_3O_4$	5	3.68	Yes	*Adv. Funct. Mater. (2023)[S87]*
	FeSiAl	2.8	5.9	No	*Adv. Funct. Mater. (2023)[S88]*
	Graphene@ $FeGeTe_2/FeTe_2/Fe_3Ge$	2.5	5.8	Yes	*Adv. Funct. Mater. (2023)*[S89]
Co containing absorbers	$Ti_3C_2T_x$@ZIF-67(Co)	2	4.8	Yes	*Adv. Funct. Mater. (2023)[S90]*
	$Co_{1-x}S$	5.6	2.5	Yes	*Adv. Funct. Mater. (2018)[S91]*
	Graphene@Co	2.3	6.21	Yes	*Adv. Funct. Mater. (2023)[S92]*
	$CuCo_2S_4$@Expanded Graphite	1.4	4.14	Yes	*Nat. Commun. (2023)[S13]*
Ni containing absorbers	Ni fibers	2.2	4.12	Yes	*Adv. Funct. Mater. (2023)[S93]*
	$Ti_3C_2T_x$@Ni	1.5	4.48	Yes	*ACS Nano (2021)[S94]*
	$Ti_3C_2T_x$@ZnO@Ni	2.8	4	Yes	*ACS Nano (2020)[S95]*
	Ni-Single atoms3/N-doped carbon	2.5	7.08	Yes	*Adv. Funct. Mater. (2022)[S96]*
	$Cu_{1.3}Ni_{1.7}(HITP)_2$	2.1	6.16	Yes	*Adv. Funct. Mater. (2023)[S97]*
Ag containing absorbers	Ga-Ag	1.4	3.6	No	*Adv. Funct. Mater. (2023)[S17]*
	AgNW@C/Co	3	1.5	Yes	*Small (2023)[S98]*
	Ag/AgCl/PANI/GO	2.63	4.77	Yes	*ACS Appl. Nano Mater. (2023)[S16]*
	AgNWs/Ti_3SiC_2	2	3.22	No	*J. Alloy. Compd. (2022)[S99]*
	AgNWs@Ni	2.93	4.06	No	*J. Colloid Interf. Sci (2023)[S100]*
	AgNWs/FeNWs	2.5	6.37	No	*J. Colloid Interf. Sci (2024)[S101]*
Others	Mo_xC	1.6	3	Yes	*Adv. Funct. Mater. (2023)[S102]*
	$(Ca_{0.2}Sr_{0.2}Ba_{0.2}La_{0.2}Pb_{0.2})TiO_3$	2	1.4	Yes	*Adv. Mater. (2023)[S103]*
	**AgNW by-products**	**2.4**	**6.7**	**No**	**This work**
	**Recycled AgNW by-products (Low conductivity)**	**3**	**8.2**	**No**	

Table S6 EMI shielding and microwave absorption performance of dual electromagnetic functional composites

Type	Filler	Content [wt%]*	EMI SE [dB]	RL_{min} [dB]	EAB [GHz]	Refs.
Fe containing composites	$Fe/Fe_3C@C$	20	8	-56.4	4.8	*J. Phys. D: Appl. Phys. (2022)[S104]*
		30	19	-12	1.71	
	$NiO@NiFe_2O_4/rGO$	40	6	-55	6.4	*Small (2023)[S105]*
		50	7.5	-18.3	5	
	Fe/C	25	8	-56.8	3.6	*Carbon (2023)[S106]*
		65	15	-7.1	0	
C containing composites	CNT	0.04 vol%	9	-61	4.3	*Mater. Today Phys. (2022)[S107]*
	CNT	0.53 vol%	\	-18	5.8	*Carbon (2020)[S108]*
		3.0 vol%	20	\	\	
	CNT/Activated carbon	10	28	-12	1.5	*Carbon (2022)[S109]*
		25	137	-2.5	0	
	Cotton fiber@C/Co	20 (600 °C)	2	-58.8	2.3	*Carbon (2023)[S110]*
		20 (800 °C)	42	-2	0	
	WSe@CNT	50	6	-61.6	2.32	*Appl. Surf. Sci. (2022)[S111]*
		90	17	-6	0	
	$Ti_3C_2T_x$/Ni Chain	10	3.5	-49.9	2.1	*ACS Appl. Mater. Interfaces (2019)[S112]*
		30	22	-8.7	0	
Others	$NiCo_2S_4$	20	9	-49.1	\	*J. Phys. D: Appl. Phys. (2018)[S113]*
		40	25	-6	0	
	PPy	\	40	-12.5	4.2	*ACS Appl. Mater. Interfaces (2023)[S114]*
	Ti_3AlC_2/SiC_β	\	12	-42.8	2.7	*ACS Appl. Electron. Mater. (2023)[S115]*
	AgNW@C/Co	30 (More Co)	26	-44.5	0.53	*Small (2023)[S98]*
		30 (Less Co)	30	-6	0	
	**AgNW By-products**	**1.3**	**34**	**-35**	**6.7**	**This work**

1. Values of EMI SE indicate maximum value in measured range.
2. The blue number indicates that the requirement of EMI shielding effectiveness (>20 dB) or reflect loss (<10 dB) is not met.

Table S7 Comparison of properties of nanosilver-based materials

	AgNPs	AWBps	AgNWs
Component dimension(s)	0D	**0D, quasi-1D, 1D**	1D
Percolation threshold [wt%]	Ultrahigh (~40)[S116]	**2.7**	Ultralow (~0.2)
Heterointerfaces	\	**Ag/AgBr and Ag/AgCl**	\
Condition of forming self-standing film/foam	Matrix	**Matrix**	Without extra matrix
Forming self-standing film (<0.1 mm)?	No	**Yes**	No
Flexibility (bend)	Good	**Excellent**	Excellent
Flexibility(stretchability)	Poor	**Good**	Excellent
Gradient structures?	No	**Yes**	No
Microwave absorption (MA)?	No	**Yes**	Basically no
Effective absorption bandwidth (EAB)?	\	**Large (~6.7 GHz)**	Normal (~4.8 GHz)
Reflective loss (RL_{min})	\	**-35 dB**	-16.5 dB~-23.9 dB
Dual EM functions (EMI shielding and MA)?	No	**Yes**	Basically no
Manufacture difficulty level	Moderate	**Easy**	difficult
Manufacture characteristics	Mass production	**From "waste"**	Complicated, more by-products

Supplementary References

[S1] S. Deville, E. Saiz, R. K. Nalla, A. P. Tomsia. Freezing as a path to build complex composites. Science **311**(5760), 515-518 (2006). https://doi.org/10.1126/science.1120937

[S2] Y. Han, K. Ruan, J. Gu. Multifunctional thermally conductive composite films based on fungal tree-like heterostructured silver nanowires@boron nitride nanosheets and aramid nanofibers. Angew. Chem. Int. Edit. **62**(5), e202216093 (2023). https://doi.org/10.1002/anie.202216093

[S3] J. S. Griffin, M. F. Bertino, T. M. Selden, S. M. Członka, S. A. Steiner. in Freeze drying. ed.by AEGERTER M A, LEVENTIS N, KOEBEL M, et al. (Springer International Publishing; Cham, 2023), pp. 121-131.

[S4] J. Feng, B.-L. Su, H. Xia, S. Zhao, C. Gao et al., Printed aerogels: Chemistry, processing,

and applications. Chem. Soc. Rev. **50**(6), 3842-3888 (2021). https://doi.org/10.1039/C9CS00757A

[S5] N. Hüsing, U. Schubert. Aerogels—airy materials: Chemistry, structure, and properties. Angew. Chem. Int. Edit. **37**(1-2), 22-45 (1998).

[S6] N. Wu, Y. Yang, C. Wang, Q. Wu, F. Pan et al., Ultrathin cellulose nanofiber assisted ambient-pressure-dried, ultralight, mechanically robust, multifunctional Mxene aerogels. Adv. Mater. **35**(1), 2207969 (2023). https://doi.org/10.1002/adma.202207969

[S7] S. S. Kistler. Coherent expanded aerogels and jellies. Nature **127**(3211), 741-741 (1931). https://doi.org/10.1038/127741a0

[S8] A. Hunt, R. Ewing, B. Ghanbarian. Percolation theory for flow in porous media. (Springer; 2014).

[S9] D. C. Kim, H. J. Shim, W. Lee, J. H. Koo, D.-H. Kim. Material-based approaches for the fabrication of stretchable electronics. Adv. Mater. **32**(15), 1902743 (2020). https://doi.org/10.1002/adma.201902743

[S10] X. Yue, H.-B. Yang, Z.-M. Han, Y.-X. Lu, C.-H. Yin et al., Tough and moldable sustainable cellulose-based structural materials via multiscale interface engineering. Adv. Mater. 2306451 (2023). https://doi.org/10.1002/adma.202306451

[S11] X. Yue, H.-B. Yang, Z.-M. Han, Y.-X. Lu, C.-H. Yin et al., Tough and moldable sustainable cellulose-based structural materials via multiscale interface engineering. Adv. Mater. 2306451 (2023). https://doi.org/10.1002/adma.202306451

[S12] Y. Chen, L. Zhang, Y. Yang, B. Pang, W. Xu et al., Recent progress on nanocellulose aerogels: Preparation, modification, composite fabrication, applications. Adv. Mater. **33**(11), 2005569 (2021). https://doi.org/10.1002/adma.202005569

[S13] Z. Tang, L. Xu, C. Xie, L. Guo, L. Zhang et al., Synthesis of $CuCo_2S_4$@expanded graphite with crystal/amorphous heterointerface and defects for electromagnetic wave absorption. Nat. Commun. **14**(1), (2023). https://doi.org/10.1038/s41467-023-41697-6

[S14] Y. Wang, H. Qin, Z. Li, J. Dai, H.-P. Cong et al., Highly compressible and environmentally adaptive conductors with high-tortuosity interconnected cellular architecture. Nat. Synth. **1**(12), 975-986 (2022). https://doi.org/10.1038/s44160-022-00167-5

[S15] B. Zhao, Y. Du, H. Lv, Z. Yan, H. Jian et al., Liquid-metal-assisted programmed galvanic engineering of core–shell nanohybrids for microwave absorption. Adv. Funct. Mater. **33**(34), 2302172 (2023). https://doi.org/10.1002/adfm.202302172

[S16] W. Fu, W. Yang, C. Qian, Y. Fu, Y. Zhu. One-pot synthesis of Ag/AgCl heterojunction nanoparticles on polyaniline nanocone arrays on graphene oxide for microwave absorption. ACS Appl. Nano Mater. **6**(5), 3728-3737 (2023). https://doi.org/10.1021/acsanm.2c05440

[S17] B. Zhao, Y. Du, H. Lv, Z. Yan, H. Jian et al., Liquid-metal-assisted programmed galvanic engineering of core–shell nanohybrids for microwave absorption. Adv. Funct. Mater. **33**(34), 2302172 (2023). https://doi.org/10.1002/adfm.202302172

[S18] Z. Wu, H.-W. Cheng, C. Jin, B. Yang, C. Xu et al., Dimensional design and core–shell engineering of nanomaterials for electromagnetic wave absorption. Adv. Mater. **34**(11), 2107538 (2022). https://doi.org/10.1002/adma.202107538

[S19] Y. Bhattacharjee, S. Bose. Core–shell nanomaterials for microwave absorption and electromagnetic interference shielding: A review. ACS Appl. Nano Mater. **4**(2), 949-972 (2021). https://doi.org/10.1021/acsanm.1c00278

[S20] C. Chen, Y. Zhao, W. Wei, J. Tao, G. Lei et al., Fabrication of silver nanowire transparent conductive films with an ultra-low haze and ultra-high uniformity and their application in transparent electronics. J. Mater. Chem. C **5**(9), 2240-2246 (2017). https://doi.org/10.1039/C6TC05455B

[S21] Y. Sun, Y. Su, Z. Chai, L. Jiang, L. Heng. Flexible solid-liquid bi-continuous electrically and thermally conductive nanocomposite for electromagnetic interference shielding and heat dissipation. Nat. Commun. **15**(1), 7290 (2024). https://doi.org/10.1038/s41467-024-51732-9

[S22] Y. Hou, Z. Sheng, C. Fu, J. Kong, X. Zhang. Hygroscopic holey graphene aerogel fibers enable highly efficient moisture capture, heat allocation and microwave absorption. Nat. Commun. **13**(1), (2022). https://doi.org/10.1038/s41467-022-28906-4

[S23] J. J. Patil, W. H. Chae, A. Trebach, K.-J. Carter, E. Lee et al., Failing forward: Stability of transparent electrodes based on metal nanowire networks. Adv. Mater. **33**(5), 2004356 (2021). https://doi.org/10.1002/adma.202004356

[S24] J. R. Greer, W. D. Nix. Nanoscale gold pillars strengthened through dislocation starvation. Phys. Rev. B **73**(24), 245410 (2006). https://doi.org/10.1103/PhysRevB.73.245410

[S25] Z. Wu, Y.-W. Zhang, M. H. Jhon, H. Gao, D. J. Srolovitz. Nanowire failure: Long = brittle and short = ductile. Nano Lett. **12**(2), 910-914 (2012). https://doi.org/10.1021/nl203980u

[S26] S. Narayanan, G. Cheng, Z. Zeng, Y. Zhu, T. Zhu. Strain hardening and size effect in five-fold twinned Ag nanowires. Nano Lett. **15**(6), 4037-4044 (2015). https://doi.org/10.1021/acs.nanolett.5b01015

[S27] J. Y. Jung, N. Qaiser, G. Feng, B.-i. Hwang, T. Kim et al., Size-dependent hardness of five-fold twin structured Ag nanowires. Phys. Chem. Chem. Phys. **19**(2), 1311-1319 (2017). https://doi.org/10.1039/C6CP07424C

[S28] H.-Y. Kim, J.-Y. Kim, K.-T. Yoo, W.-J. Yang, J.-W. Byeon. Failure mechanism of Ag nanowire-coated conductive transparent electrode for wearable devices under folding and torsional fatigue condition. Microelectronics Reliability. **88-90**, 345-349 (2018). https://doi.org/10.1016/j.microrel.2018.07.120

[S29] J. Zhao, H. Sun, S. Dai, Y. Wang, J. Zhu. Electrical breakdown of nanowires. Nano Lett. **11**(11), 4647-4651 (2011). https://doi.org/10.1021/nl202160c

[S30] C. Ma, T. Mai, P.-L. Wang, W.-Y. Guo, M.-G. Ma. Flexible MXene/nanocellulose composite aerogel film with cellular structure for electromagnetic interference shielding and photothermal conversion. ACS Appl. Mater. Interfaces **15**(40), 47425-47433 (2023). https://doi.org/10.1021/acsami.3c12171

[S31] W. Xin, M.-G. Ma, F. Chen. Silicone-coated mxene/cellulose nanofiber aerogel films with photothermal and joule heating performances for electromagnetic interference shielding. ACS Appl. Nano Mater. **4**(7), 7234-7243 (2021). https://doi.org/10.1021/acsanm.1c01185

[S32] J. Wang, Q. Li, K. Li, X. Sun, Y. Wang et al., Ultra-high electrical conductivity in filler-free polymeric hydrogels toward thermoelectrics and electromagnetic interference shielding. Adv. Mater. **34**(12), 2109904 (2022). https://doi.org/10.1002/adma.202109904

[S33] J. Zhang, W. Guo, S. Shen, Q. Zhang, X. Chen et al., High-compressive, elastic, and wearable cellulose nanofiber-based carbon aerogels for efficient electromagnetic interference shielding. ACS Appl. Mater. Interfaces **16**(13), 16612-16621 (2024). https://doi.org/10.1021/acsami.3c16559

[S34] Z. Zeng, T. Wu, D. Han, Q. Ren, G. Siqueira et al., Ultralight, flexible, and biomimetic nanocellulose/silver nanowire aerogels for electromagnetic interference shielding. ACS Nano **14**(3), 2927-2938 (2020). https://doi.org/10.1021/acsnano.9b07452

[S35] L. Feng, P. Wei, Q. Song, J. Zhang, Q. Fu et al., Superelastic, highly conductive, superhydrophobic, and powerful electromagnetic shielding hybrid aerogels built from orthogonal graphene and boron nitride nanoribbons. ACS Nano **16**(10), 17049-17061 (2022). https://doi.org/10.1021/acsnano.2c07187

[S36] M. Han, X. Yin, K. Hantanasirisakul, X. Li, A. Iqbal et al., Anisotropic MXene aerogels with a mechanically tunable ratio of electromagnetic wave reflection to absorption. Adv. Opt. Mater. **7**(10), 1900267 (2019). https://doi.org/10.1002/adom.201900267

[S37] S. Wu, M. Zou, Z. Li, D. Chen, H. Zhang et al., Robust and stable cu nanowire@graphene core–shell aerogels for ultraeffective electromagnetic interference shielding. Small **14**(23), 1800634 (2018). https://doi.org/10.1002/smll.201800634

[S38] Y. Xie, S. Liu, K. Huang, B. Chen, P. Shi et al., Ultra-broadband strong electromagnetic interference shielding with ferromagnetic graphene quartz fabric. Adv. Mater. **34**(30), 2202982 (2022). https://doi.org/10.1002/adma.202202982

[S39] Q. Wei, S. Pei, X. Qian, H. Liu, Z. Liu et al., Superhigh electromagnetic interference shielding of ultrathin aligned pristine graphene nanosheets film. Adv. Mater. **32**(14), 1907411 (2020). https://doi.org/10.1002/adma.201907411

[S40] Q.-F. Guan, Z.-M. Han, K.-P. Yang, H.-B. Yang, Z.-C. Ling et al., Sustainable double-network structural materials for electromagnetic shielding. Nano Lett. **21**(6), 2532-2537 (2021). https://doi.org/10.1021/acs.nanolett.0c05081

[S41] C. Fu, Z. Sheng, X. Zhang. Laminated structural engineering strategy toward carbon nanotube-based aerogel films. ACS Nano **16**(6), 9378-9388 (2022). https://doi.org/10.1021/acsnano.2c02193

[S42] Z. Zeng, H. Jin, M. Chen, W. Li, L. Zhou et al., Lightweight and anisotropic porous MWCNT/WPU composites for ultrahigh performance electromagnetic interference shielding. Adv. Funct. Mater. **26**(2), 303-310 (2016). https://doi.org/10.1002/adfm.201503579

[S43] Y. Chen, H.-B. Zhang, Y. Yang, M. Wang, A. Cao et al., High-performance epoxy nanocomposites reinforced with three-dimensional carbon nanotube sponge for electromagnetic interference shielding. Adv. Funct. Mater. **26**(3), 447-455 (2016). https://doi.org/10.1002/adfm.201503782

[S44] P. Hu, J. Lyu, C. Fu, W.-b. Gong, J. Liao et al., Multifunctional aramid nanofiber/carbon nanotube hybrid aerogel films. ACS Nano **14**(1), 688-697 (2019). https://doi.org/10.1021/acsnano.9b07459

[S45] Q. Song, F. Ye, X. Yin, W. Li, H. Li et al., Carbon nanotube–multilayered graphene edge plane core–shell hybrid foams for ultrahigh-performance electromagnetic-interference shielding. Adv. Mater. **29**(31), 1701583 (2017). https://doi.org/10.1002/adma.201701583

[S46] X. Liu, Y. Li, X. Sun, W. Tang, G. Deng et al., Off/on switchable smart electromagnetic interference shielding aerogel. Matter **4**(5), 1735-1747 (2021). https://doi.org/10.1016/j.matt.2021.02.022

[S47] X. Ma, J. Pan, H. Guo, J. Wang, C. Zhang et al., Ultrathin wood-derived conductive carbon composite film for electromagnetic shielding and electric heating management. Adv. Funct. Mater. **33**(16), 2213431 (2023). https://doi.org/10.1002/adfm.202213431

[S48] L. Zhuang, D. Lu, J. Zhang, P. Guo, L. Su et al., Highly cross-linked carbon tube aerogels with enhanced elasticity and fatigue resistance. Nat. Commun. **14**(1), (2023). https://doi.org/10.1038/s41467-023-38664-6

[S49] Y. Zhang, K. Ruan, K. Zhou, J. Gu. Controlled distributed $Ti_3C_2T_x$ hollow microspheres on thermally conductive polyimide composite films for excellent electromagnetic interference shielding. Adv. Mater. **35**(16), 2211642 (2023). https://doi.org/10.1002/adma.202211642

[S50] H. Wang, R. Lu, J. Yan, J. Peng, A. P. Tomsia et al., Tough and conductive nacre-inspired MXene/epoxy layered bulk nanocomposites. Angew. Chem. Int. Edit. **62**(9), e202216874 (2023). https://doi.org/10.1002/anie.202216874

[S51] R. Sun, H.-B. Zhang, J. Liu, X. Xie, R. Yang et al., Highly conductive transition metal carbide/carbonitride(MXene)@polystyrene nanocomposites fabricated by electrostatic assembly for highly efficient electromagnetic interference shielding. Adv. Funct. Mater. **27**(45), 1702807 (2017). https://doi.org/10.1002/adfm.201702807

[S52] Q.-W. Wang, H.-B. Zhang, J. Liu, S. Zhao, X. Xie et al., Multifunctional and water-resistant MXene-decorated polyester textiles with outstanding electromagnetic interference shielding and joule heating performances. Adv. Funct. Mater. **29**(7), 1806819 (2019). https://doi.org/10.1002/adfm.201806819

[S53] L.-X. Liu, W. Chen, H.-B. Zhang, Q.-W. Wang, F. Guan et al., Flexible and multifunctional silk textiles with biomimetic leaf-like MXene/silver nanowire nanostructures for electromagnetic interference shielding, humidity monitoring, and self-derived hydrophobicity. Adv. Funct. Mater. **29**(44), 1905197 (2019). https://doi.org/10.1002/adfm.201905197

[S54] Z. Ma, S. Kang, J. Ma, L. Shao, Y. Zhang et al., Ultraflexible and mechanically strong double-layered aramid nanofiber–$Ti_3C_2T_x$ MXene/silver nanowire nanocomposite papers for high-performance electromagnetic interference shielding. ACS Nano 14(7), 8368-8382 (2020). https://doi.org/10.1021/acsnano.0c02401

[S55] M. Cheng, M. Ying, R. Zhao, L. Ji, H. Li et al., Transparent and flexible electromagnetic interference shielding materials by constructing sandwich AgNW@MXene/wood composites. ACS Nano **16**(10), 16996-17007 (2022). https://doi.org/10.1021/acsnano.2c07111

[S56] A. Ghaffarkhah, S. A. Hashemi, S. Rostami, M. Amini, F. Ahmadijokani et al., Ultra-flyweight cryogels of MXene/graphene oxide for electromagnetic interference shielding. Adv. Funct. Mater. 2304748 (2023). https://doi.org/10.1002/adfm.202304748

[S57] Z. Wang, X. Xia, M. Zhu, X. Zhang, R. Liu et al., Rational assembly of liquid metal/elastomer lattice conductors for high-performance and strain-invariant stretchable electronics. Adv. Funct. Mater. **32**(10), 2108336 (2022). https://doi.org/10.1002/adfm.202108336

[S58] B. Yao, W. Hong, T. Chen, Z. Han, X. Xu et al., Highly stretchable polymer composite with strain-enhanced electromagnetic interference shielding effectiveness. Adv. Mater. **32**(14), 1907499 (2020). https://doi.org/10.1002/adma.201907499

[S59] S. Lee, N. K. Nguyen, W. Kim, M. Kim, V. A. Cao et al., Absorption-dominant electromagnetic interference shielding through electrical polarization and triboelectrification in surface-patterned ferroelectric poly[(vinylidenefluoride-*co*-trifluoroethylene)-MXene] composite. Adv. Funct. Mater. 2307588 (2023). https://doi.org/10.1002/adfm.202307588

[S60] C. Liang, Y. Liu, Y. Ruan, H. Qiu, P. Song et al., Multifunctional sponges with flexible motion sensing and outstanding thermal insulation for superior electromagnetic interference shielding. Compos. Pt. A-Appl. Sci. Manuf. **139**, 106143 (2020). https://doi.org/10.1016/j.compositesa.2020.106143

[S61] Y.-y. Xiao, Y.-j. He, R.-q. Wang, Y.-z. Lei, J.-h. Yang et al., Mussel-inspired strategy to construct 3D silver nanoparticle network in flexible phase change composites with excellent thermal energy management and electromagnetic interference shielding capabilities. Compos. Pt. B-Eng. **239**, 109962 (2022). https://doi.org/10.1016/j.compositesb.2022.109962

[S62] J. Dong, Y. Peng, J. Long, Y. Zhang, Z. Wang et al., An all-stretchable, ultraviolet protective, and electromagnetic-interference-free e-textile. Adv. Funct. Mater. 2308426 (2023). https://doi.org/10.1002/adfm.202308426

[S63] W. Ren, H. Zhu, Y. Yang, Y. Chen, H. Duan et al., Flexible and robust silver coated non-woven fabric reinforced waterborne polyurethane films for ultra-efficient electromagnetic shielding. Compos. Pt. B-Eng. **184**, 107745 (2020). https://doi.org/10.1016/j.compositesb.2020.107745

[S64] Q. Zhou, J. Lyu, G. Wang, M. Robertson, Z. Qiang et al., Mechanically strong and multifunctional hybrid hydrogels with ultrahigh electrical conductivity. Adv. Funct. Mater. **31**(40), 2104536 (2021). https://doi.org/10.1002/adfm.202104536

[S65] Z. Ma, X. Xiang, L. Shao, Y. Zhang, J. Gu. Multifunctional wearable silver nanowire decorated leather nanocomposites for joule heating, electromagnetic interference shielding and piezoresistive sensing. Angew. Chem. Int. Edit. **61**(15), e202200705 (2022). https://doi.org/10.1002/anie.202200705

[S66] B. Yuan, S. Lai, J. Li, L. Li, S. Bai et al., Trash into treasure: Stiff, thermally insulating and highly conductive carbon aerogels from leather wastes for high-performance electromagnetic interference shielding. J. Mater. Chem. C **9**(7), 2298-2310 (2021). https://doi.org/10.1039/D0TC05480A

[S67] S. Zeng, Z.-x. Huang, H. Jiang, Y. Li. From waste to wealth: A lightweight and flexible leather solid waste/polyvinyl alcohol/silver paper for highly efficient electromagnetic interference shielding. ACS Appl. Mater. Interfaces **12**(46), 52038-52049 (2020). https://doi.org/10.1021/acsami.0c16169

[S68] H. Shi, M. Jiang, T. Zhang, S. Chen, L. Li et al., Multifunctional silver decorated leather solid waste/poly(vinyl alcohol) nanocomposites for electromagnetic interference shielding, joule heating and crude-oil cleaning. ACS Sustain. Chem. Eng. **10**(39), 13165-13175 (2022). https://doi.org/10.1021/acssuschemeng.2c04229

[S69] T. Zhang, S. Zeng, H. Jiang, Z. Li, D. Bai et al., Leather solid waste/poly(vinyl alcohol)/polyaniline aerogel with mechanical robustness, flame retardancy, and enhanced

electromagnetic interference shielding. ACS Appl. Mater. Interfaces **13**(9), 11332-11343 (2021). https://doi.org/10.1021/acsami.1c00880

[S70] D. Gao, S. Guo, Y. Zhou, B. Lyu, X. Li et al., Absorption-dominant, low-reflection multifunctional electromagnetic shielding material derived from hydrolysate of waste leather scraps. ACS Appl. Mater. Interfaces **14**(33), 38077-38089 (2022). https://doi.org/10.1021/acsami.2c10787

[S71] D. Gao, S. Guo, Y. Zhou, B. Lyu, J. Ma et al., Hydrophobic, flexible electromagnetic interference shielding films derived from hydrolysate of waste leather scraps. J. Colloid Interf. Sci. **613**, 396-405 (2022). https://doi.org/10.1016/j.jcis.2022.01.043

[S72] X. Peng, X. Meng, B. Yu, H. Chen, Z. Liu et al., Graphitized and flexible porous textile updated from waste cotton for wearable electromagnetic interference shielding. Carbon. **207**, 144-153 (2023). https://doi.org/10.1016/j.carbon.2023.02.044

[S73] Y. Zhang, G. Shen, S. S. Lam, S. Ansar, S.-C. Jung et al., A waste textiles-based multilayer composite fabric with superior electromagnetic shielding, infrared stealth and flame retardance for military applications. Chem. Eng. J. **471**, 144679 (2023). https://doi.org/10.1016/j.cej.2023.144679

[S74] X. Ma, B. Shen, L. Zhang, Z. Chen, Y. Liu et al., Novel straw-derived carbon materials for electromagnetic interference shielding: A waste-to-wealth and sustainable initiative. ACS Sustain. Chem. Eng. **7**(10), 9663-9670 (2019). https://doi.org/10.1021/acssuschemeng.9b01288

[S75] H. Wang, R. Xu, L. Dong, X. Zhang, D. Tu et al., Development of biodegradable and low-cost electromagnetic shielding composite by waste porous biochar and poly (butylene succinate). Polym. Composite **44**(9), 6049-6070 (2023). https://doi.org/10.1002/pc.27546

[S76] S. Raji, G. K. Sharma, B. R. Aranya, K. Prabhakaran. Carbon composite foams from the wasted banana leaf for EMI shielding and thermal insulation. Carbon. **213**, 118259 (2023). https://doi.org/10.1016/j.carbon.2023.118259

[S77] E. Pakdel, S. Kashi, T. Baum, K. A. S. Usman, J. M. Razal et al., Carbon fibre waste recycling into hybrid nonwovens for electromagnetic interference shielding and sound absorption. J. Clean Prod. **315**, 128196 (2021). https://doi.org/10.1016/j.jclepro.2021.128196

[S78] L. Vazhayal, P. Wilson, K. Prabhakaran. Waste to wealth: Lightweight, mechanically strong and conductive carbon aerogels from waste tissue paper for electromagnetic shielding and CO_2 adsorption. Chem. Eng. J. **381**, 122628 (2020). https://doi.org/10.1016/j.cej.2019.122628

[S79] S. Liu, B. Quan, M. Sheng, Y. Yang, X. Hu et al., A novel in-situ growth ZIF-67 on biological porous carbon encapsulated phase change composites with electromagnetic interference shielding and multifunctional energy conversion. Nano Energy **114**, 108669 (2023). https://doi.org/10.1016/j.nanoen.2023.108669

[S80] R. Prasad, A. R. Pai, S. O. Oyadiji, S. Thomas, S. K. S. Parashar. Utilization of hazardous red mud in silicone rubber/MWCNT nanocomposites for high performance electromagnetic interference shielding. J. Clean Prod. **377**, 134290 (2022). https://doi.org/10.1016/j.jclepro.2022.134290

[S81] Y. Xie, P. Li, J. Tang, B. Wei, W. Chen et al., Highly thermally conductive and superior

electromagnetic interference shielding composites via in situ microwave-assisted reduction/exfoliation of expandable graphite. Compos. Pt. A-Appl. Sci. Manuf. **149**, 106517 (2021). https://doi.org/10.1016/j.compositesa.2021.106517

[S82] Y. Liu, N. Wu, S. Zheng, Y. Yang, B. Li et al., From MXene trash to ultraflexible composites for multifunctional electromagnetic interference shielding. ACS Appl. Mater. Interfaces **14**(44), 50120-50128 (2022). https://doi.org/10.1021/acsami.2c13849

[S83] Y. Yang, N. Wu, B. Li, W. Liu, F. Pan et al., Biomimetic porous MXene sediment-based hydrogel for high-performance and multifunctional electromagnetic interference shielding. ACS Nano **16**(9), 15042-15052 (2022). https://doi.org/10.1021/acsnano.2c06164

[S84] B. Li, N. Wu, Q. Wu, Y. Yang, F. Pan et al., From "100%" utilization of MAX/MXene to direct engineering of wearable, multifunctional e-textiles in extreme environments. Adv. Funct. Mater. 2307301 (2023). https://doi.org/10.1002/adfm.202307301

[S85] R. Zhao, T. Gao, Y. Li, Z. Sun, Z. Zhang et al., Highly anisotropic Fe_3C microflakes constructed by solid-state phase transformation for efficient microwave absorption. Nat. Commun. **15**(1), (2024). https://doi.org/10.1038/s41467-024-45815-w

[S86] L. Rao, L. Wang, C. Yang, R. Zhang, J. Zhang et al., Confined diffusion strategy for customizing magnetic coupling spaces to enhance low-frequency electromagnetic wave absorption. Adv. Funct. Mater. **33**(16), 2213258 (2023). https://doi.org/10.1002/adfm.202213258

[S87] R. Che, Z. Wu, B. Quan, R. Zhang, H. Zhang et al., Machine learning-directed fast and high-throughput acquisition of high-efficiency microwave absorbents from infinite design space. Adv. Funct. Mater. 2303108 (2023). https://doi.org/10.1002/adfm.202303108

[S88] M. Liu, Z. Wu, L. Yang, X. Lv, R. Zhang et al., Finite-sized atom reconstruction enhanced high-frequency multi-domain magnetic response. Adv. Funct. Mater. 2307943 (2023). https://doi.org/10.1002/adfm.202307943

[S89] G. Li, S. Ma, Z. Li, Y. Zhang, Y. Cao et al., Temperature-induced self-decomposition doping of Fe_3GeTe_2 to achieve ultra-high Tc of 496 K for multispectral compatible strong electromagnetic wave absorption. Adv. Funct. Mater. **33**(15), 2210578 (2023). https://doi.org/10.1002/adfm.202210578

[S90] P. Liu, G. Zhang, H. Xu, S. Cheng, Y. Huang et al., Synergistic dielectric–magnetic enhancement via phase-evolution engineering and dynamic magnetic resonance. Adv. Funct. Mater. **33**(13), 2211298 (2023). https://doi.org/10.1002/adfm.202211298

[S91] X.-J. Zhang, J.-Q. Zhu, P.-G. Yin, A.-P. Guo, A.-P. Huang et al., Tunable high-performance microwave absorption of co1–xs hollow spheres constructed by nanosheets within ultralow filler loading. Adv. Funct. Mater. **28**(49), 1800761 (2018). https://doi.org/10.1002/adfm.201800761

[S92] Y. Liu, X. Wei, X. He, J. Yao, R. Tan et al., Multifunctional shape memory composites for joule heating, self-healing, and highly efficient microwave absorption. Adv. Funct. Mater. **33**(5), 2211352 (2023). https://doi.org/10.1002/adfm.202211352

[S93] F. Pan, M. Ning, Z. Li, D. Batalu, H. Guo et al., Sequential architecture induced strange dielectric-magnetic behaviors in ferromagnetic microwave absorber. Adv. Funct. Mater. **33**(27), 2300374 (2023). https://doi.org/10.1002/adfm.202300374

[S94] C. Wen, X. Li, R. Zhang, C. Xu, W. You et al., High-density anisotropy magnetism enhanced microwave absorption performance in $Ti_3C_2T_x$ MXene@Ni microspheres. ACS Nano **16**(1), 1150-1159 (2021). https://doi.org/10.1021/acsnano.1c08957

[S95] S. Wang, D. Li, Y. Zhou, L. Jiang. Hierarchical MXene $Ti_3C_2T_x$ /Ni chain/ZnO array hybrid nanostructures on cotton fabric for durable self-cleaning and enhanced microwave absorption. ACS Nano **14**(7), 8634-8645 (2020). https://doi.org/10.1021/acsnano.0c03013

[S96] H. Liang, G. Chen, D. Liu, Z. Li, S. Hui et al., Exploring the Ni 3D orbital unpaired electrons induced polarization loss based on ni single-atoms model absorber. Adv. Funct. Mater. **33**(7), 2212604 (2023). https://doi.org/10.1002/adfm.202212604

[S97] C. Chen, Z. Shan, S. Tao, A. Xie, H. Yang et al., Atomic tuning in electrically conducting bimetallic organic frameworks for controllable electromagnetic wave absorption. Adv. Funct. Mater. 2305082 (2023). https://doi.org/10.1002/adfm.202305082

[S98] C. Jiang, B. Wen. Construction of 1D heterogeneous Co/C@AgNWs with tunable electromagnetic wave absorption and shielding performance. Small **19**(34), 2301760 (2023). https://doi.org/10.1002/smll.202301760

[S99] Y. Liu, J. Yang, J. Xu, L. Lu, X. Su. Electromagnetic and microwave absorption properties of Ti_3SiC_2/AgNWs/ acrylic acid resin composite coatings with FSS incorporation. J. Alloys Compounds **899**, 163327 (2022). https://doi.org/10.1016/j.jallcom.2021.163327

[S100] T. Hang, J. Zheng, Y. Zheng, S. Jiang, L. Zhou et al., Wheat-like ni-coated core–shell silver nanowires for effective electromagnetic wave absorption. J. Colloid Interf. Sci. **649**, 394-402 (2023). https://doi.org/10.1016/j.jcis.2023.06.087

[S101] T. Hang, C. Xu, J. Shen, J. Zheng, L. Zhou et al., Ultra-flexible silver/iron nanowire decorated melamine composite foams for high-efficiency electromagnetic wave absorption and thermal management. J. Colloid Interf. Sci. **654**, 945-954 (2024). https://doi.org/10.1016/j.jcis.2023.10.117

[S102] M. Yuan, H. Lv, H. Cheng, B. Zhao, G. Chen et al., Atomic and electronic reconstruction in defective 0D molybdenum carbide heterostructure for regulating lower-frequency microwaves. Adv. Funct. Mater. **33**(33), 2302003 (2023). https://doi.org/10.1002/adfm.202302003

[S103] B. Zhao, Z. Yan, Y. Du, L. Rao, G. Chen et al., High-entropy enhanced microwave attenuation in titanate perovskites. Adv. Mater. **35**(11), 2210243 (2023). https://doi.org/10.1002/adma.202210243

[S104] X. Yang, Y. Zhou, H. Xing, H. Wang, W. Feng et al., MIL-88B (Fe) driven Fe/Fe3c encapsulated in high-crystalline carbon for high-efficient microwave absorption and electromagnetic interference shielding. J. Phys. D: Appl. Phys. **55**(14), 145003 (2022). https://doi.org/10.1088/1361-6463/ac3e29

[S105] L. Yao, Y. Wang, J. Zhao, Y. Zhu, M. Cao. Multifunctional nanocrystalline-assembled porous hierarchical material and device for integrating microwave absorption, electromagnetic interference shielding, and energy storage. Small 2208101 (2023). https://doi.org/10.1002/smll.202208101

[S106] Q. Zheng, J. Wang, M. Yu, W.-Q. Cao, H. Zhai et al., Heterodimensional structure porous nanofibers embedded confining magnetic nanocrystals for electromagnetic functional material

and device. Carbon **210**, 118049 (2023). https://doi.org/10.1016/j.carbon.2023.118049

[S107] G. Wang, D. Yi, X. Jia, J. Chen, B. Shen et al., Structural design of compressible shape-memory foams for smart self-fixable electromagnetic shielding with reduced reflection. Mater. Today Phys. **22**, 100612 (2022). https://doi.org/10.1016/j.mtphys.2022.100612

[S108] K. Huang, M. Chen, G. He, X. Hu, W. He et al., Stretchable microwave absorbing and electromagnetic interference shielding foam with hierarchical buckling induced by solvent swelling. Carbon. **157**, 466-477 (2020). https://doi.org/10.1016/j.carbon.2019.10.059

[S109] P. Negi, A. Gupta, M. Singh, R. Kumar, S. Kumar et al., Excellent microwave absorbing and electromagnetic shielding performance of grown MWCNT on activated carbon bifunctional composite. Carbon **198**, 151-161 (2022). https://doi.org/10.1016/j.carbon.2022.07.024

[S110] J. Chen, L. Wang, B. Shen, W. Zheng. Biomass-based Co/C@carbon composites derived from mof-modified cotton fibers for enhanced electromagnetic attenuation. Carbon **210**, 118035 (2023). https://doi.org/10.1016/j.carbon.2023.118035

[S111] Y. Zhu, Q. Wang, Y. Han, L. Li, M. Cao. Constructing WSe2@CNTs heterojunction to tune attenuation capability for efficient microwave absorbing and green EMI shielding. Appl. Surf. Sci. **592**, 153253 (2022). https://doi.org/10.1016/j.apsusc.2022.153253

[S112] L. Liang, G. Han, Y. Li, B. Zhao, B. Zhou et al., Promising $Ti_3C_2T_x$ MXene/Ni chain hybrid with excellent electromagnetic wave absorption and shielding capacity. ACS Appl. Mater. Interfaces. **11**(28), 25399-25409 (2019). https://doi.org/10.1021/acsami.9b07294

[S113] W. Li, M. Zhou, F. Lu, H. Liu, Y. Zhou et al., Enhanced conductive loss in nickel–cobalt sulfide nanostructures for highly efficient microwave absorption and shielding. J. Phys. D: Appl. Phys. **51**(23), 235303 (2018). https://doi.org/10.1088/1361-6463/aac048

[S114] Y. Wang, J. Li, W. Li, L. Ma, J. Lin et al., Flexible polypyrrole-coated polyamide based mats for broadband microwave absorption and electromagnetic interference shielding with low reflection. ACS Appl. Polym. Mater. **5**(4), 2995-3004 (2023). https://doi.org/10.1021/acsapm.3c00175

[S115] P. Yao, X. Li, Y. Zhang, K. Ma, X. Zhang et al., Electromagnetic wave absorption and shielding performances and mechanisms of a porous $Ti_3C_2T_x$/SiC gradient composite. ACS Appl. Electron. Mater. **5**(3), 1558-1565 (2023). https://doi.org/10.1021/acsaelm.2c01595

[S116] E. Kim, D. Y. Lim, Y. Kang, E. Yoo. Fabrication of a stretchable electromagnetic interference shielding silver nanoparticle/elastomeric polymer composite. RSC Adv. **6**(57), 52250-52254 (2016). https://doi.org/10.1039/C6RA04765C
